# Waterborne Poly(urethane-urea)s for Lithium-Ion/Lithium-Metal Batteries

**DOI:** 10.3390/polym18020299

**Published:** 2026-01-22

**Authors:** Bushra Rashid, Anjum Hanief Kohli, In Woo Cheong

**Affiliations:** Department of Applied Chemistry, Graduate School of Chemical Engineering and Applied Chemistry, Kyungpook National University, 80 Daehak-ro, Buk-gu, Daegu 41566, Republic of Korea; bushrarashid@knu.ac.kr (B.R.); anjum2000@knu.ac.kr (A.H.K.)

**Keywords:** waterborne polyurethane, poly(urethane-urea), aqueous binder, separator coating, polymer electrolyte, lithium metal battery, ionic conductivity, adhesion, microphase separation

## Abstract

Waterborne polyurethane (WPU) and waterborne poly(urethane-urea) (WPUU) dispersions allow safer and more sustainable manufacturing of rechargeable batteries via water-based processing, while offering tunable adhesion and segmented-domain mechanics. Beyond conventional roles as binders and coatings, WPU/WPUU chemistries also support separator/interlayer and polymer-electrolyte designs for lithium-ion and lithium metal systems, where interfacial integrity, stress accommodation, and ion transport must be balanced. Here, we review WPU/WPUU fundamentals (building blocks, dispersion stabilization, morphology, and film formation) and review prior studies through a battery-centric structure–processing–property lens. We point out key performance-limiting trade-offs—adhesion versus electrolyte uptake and ionic conductivity versus storage modulus—and relate them to practical formulation variables, including soft-/hard-segment selection, ionic center/counterion design, molecular weight/topology control, and crosslinking strategies. Applications are reviewed for (i) electrode binders (graphite/Si; cathodes such as LFP and NMC), (ii) separator coatings and functional interlayers, and (iii) gel/solid polymer electrolytes and hybrid composites, with a focus on practical design guidelines for navigating these trade-offs. Future advancements in WPU/WPUU chemistries will depend on developing stable, low-impedance interlayers, enhancing electrochemical behavior, and establishing application-specific design guidelines to optimize performance in lithium metal batteries (LMB).

## 1. Introduction

Rechargeable batteries underpin electrified transportation and grid-scale storage, yet further gains in practical energy density and lifetime are increasingly limited by interfaces, mechanics, and manufacturability rather than active-material capacity alone [1]. In Lithium-ion batteries (LIB), the incumbent PVDF binder—most often processed in N-methyl-2-pyrrolidone (NMP)—carries cost, safety, and environmental burdens, and offers limited latitude to meet emerging demands such as thick, high-loading electrodes, silicon-rich anodes with large volume change, and high-voltage cathodes that exacerbate interfacial degradation [2,3]. Water-based alternatives (e.g., CMC/SBR, PAA, alginates) improve process safety but frequently face trade-offs among adhesion to current collectors/active materials, cohesive toughness under repeated strain, electrolyte compatibility, and interfacial impedance stability, especially when simultaneously targeting high loading, fast drying, and long cycle life [2,3].

WPU and WPUU offer a distinctive platform to deal with these coupled constraints because they integrate (i) water-based processing, (ii) strong, tunable adhesion/cohesion arising from segmented morphology and hydrogen-bonded hard domains, and (iii) broad formulation freedom via soft-segment chemistry, ionic/nonionic stabilization, chain extension, and network design [4,5]. In this review, we use WPU as a general term for WPU dispersions. In contrast, WPUU denotes dispersions in which urea linkages are introduced via diamine chain extension, often strengthening hydrogen bonding and hard-domain organization and thereby expanding the accessible mechanical/adhesive design space. Unlike many aqueous binder systems in which mechanical reinforcement and interfacial chemistry are tightly coupled, WPU/WPUU chemistries can more independently tune elasticity, toughness, adhesion, and polarity/ionicity, allowing electrode architectures that better tolerate stress, maintain particle contact, and suppress cracking or delamination during cycling across multiple chemistries [4,5].

Despite these advantages, translating WPU/WPUU into battery components is challenging. Waterborne processing introduces moisture-management constraints (residual water, low-molecular-weight extractables, drying history), while ionic centers used for dispersion stabilization can increase hygroscopicity and carbonate-electrolyte swelling, potentially altering interfacial resistance and dimensional stability [6,7]. Moreover, when WPU-derived networks are used as polymer electrolytes or interlayers, they inherit the classic coupling between ion transport and mechanical stiffness, whereas in binders/coatings they must balance adhesion versus electrolyte uptake to maintain low impedance without sacrificing cohesion [6,7]. Consequently, while the literature is expanding rapidly, cross-study comparisons remain challenging because processing histories and reporting depth vary widely (e.g., residual moisture control, electrolyte uptake/swelling, interfacial impedance measurement details, and mechanical benchmarks in relevant solvation states).

This review examines WPU, WPUU, and their derived films/networks as functional materials for rechargeable batteries in three representative roles: (i) electrode binders, (ii) separator coatings and functional interlayers, and (iii) gel/solid polymer electrolytes, including hybrid composite electrolytes. The main focus is a battery-centric synthesis of the governing trade-offs and practical design variables that recur across WPU/WPUU-allowed binders, separator coatings/interlayers, and polymer electrolytes. At the molecular and network level, soft-segment identity/molecular weight, hard-segment and urea contents, ionic-center type/density with counterion selection, and chain extension/crosslinking/topology control collectively govern Tg, microphase separation, adhesion/toughness, electrolyte uptake and swelling, and Li^+^ coordination environments. At the dispersion and film-formation level, solids content, pH, particle size distribution, and rheology determine coatability and microstructure development, while drying protocols govern residual moisture and interphase compatibility. For composite formulations, filler–matrix compatibility and percolation further shape mechanical reinforcement and, particularly in electrolytes, transport pathways.

Battery-relevant response metrics differ by component. For binders and coatings, we emphasize adhesion (including peel strength), cohesion/toughness, electrolyte uptake/swelling, and the evolution of interfacial resistance under realistic electrolyte exposure. For polymer electrolytes, we focus on the ion conductivity(σ)–storage modulus(E′) trade-off, Li^+^ transference number (t^+^), electrochemical stability window, and interfacial impedance behavior during cycling. To help reproducibility and meaningful comparison, we point out reporting gaps that most strongly hinder comparability (e.g., moisture/extractables management, uptake/swelling protocols, interfacial impedance measurement details, and mechanics reported in relevant solvation states) and recommend that future studies report these items consistently.

## 2. Fundamentals of Waterborne PU

### 2.1. Raw Materials

WPUs are typically synthesized by dispersing an isocyanate-terminated prepolymer in water, followed by chain extension [4,8,9]. The prepolymer backbone is mainly composed of polyol and diisocyanate. The polyol-derived soft segment constitutes most of the chain length and largely dictates Tg, segmental mobility, film formation, and interfacial wetting (via soft-phase polarity), whereas the hard segment formed by the diisocyanate and chain extender primarily governs modulus, cohesion, and thermal resistance [9,10]. In binder and coating formulations, polyol selection therefore serves as the central design handle to tune flexibility and viscoelasticity, resistance to water and solvents, and substrate-dependent adhesion, which in turn determine battery-relevant interfacial property and durability [10,11].

#### 2.1.1. Polyols

Major polyol classes provide distinct structure–property trade-offs [9,10,12]. Polyether polyols (PPG, PTMG, PEG) offer low Tg and high flexibility, benefiting film formation and tack/peel, but can reduce shear and creep resistances, and show limited oxidative stability at high voltage [12,13]. Swelling in carbonate electrolytes must be controlled via composition and crosslinking [13,14]. Polyester polyols (e.g., adipate/succinate/phthalate with BDO, NPG) increase polarity and strength and often improve adhesion to inorganic particles/current collectors, yet ester linkages remain susceptible to hydrolysis/chemical attack under harsh conditions [9,10]. Polycarbonate polyols can offer improved oxidative stability and dimensional stability that help preserve particle–binder interfaces, although their rigidity can suppress segmental mobility and ionic conductivity unless blended with ether-rich components [15,16]. PCL diols contribute creep and abrasion resistances through semi-crystallinity but may constrain ionic transport and low-temperature flexibility if crystallinity is excessive, motivating pairing with more amorphous polyols [12,17]. Specialty polyols are used to target specific functions—fluorinated polyols lower surface energy and improve repellency but can weaken wetting and adhesion (thus used sparingly or surface-targeted); acrylic polyols allow broad Tg or polarity tuning (e.g., MMA, styrene, BA/EHA) and can improve mechanical strength and crack resistance, but often require combination with softer, ion-conductive segments; rubber-like polyols (HTNR, HTPB, and bio-derived analogs) accommodate large volume changes but must be formulated to reduce swelling and adhesion loss [10,18,19,20]. Branched polyols (f ≈ 3–8) raise network density and stiffness but can introduce brittleness if not balanced [10,21]. Table 1 summarizes the relationships among polyol type, characteristic structure, key advantages, and battery-relevant considerations [11,12].

#### 2.1.2. Isocyanates

Isocyanates define polyurethane hard segments by forming urethane (with alcohols) and urea (with amines) linkages, thereby governing microphase separation and the resulting thermo-mechanical behavior [9,10]. Commercially, isocyanates are broadly categorized as aliphatic, aromatic, and polyfunctional grades, offering distinct balances among reactivity, weathering stability, and network formation [9]. Aliphatic diisocyanates (e.g., HDI, IPDI, H_12_MDI) typically provide superior UV/yellowing resistance and longer processing latitude due to lower NCO reactivity, making them attractive for durable WPU films and battery-facing formulations where stability is prioritized [10]. Aromatic diisocyanates (e.g., TDI, MDI) react rapidly and yield stiff, cohesive hard domains, but they are more prone to yellowing/photo-oxidation and can suffer pronounced NCO–water side reactions (CO_2_ foaming/branching/gelation), so strict moisture control and process choice (e.g., acetone/prepolymer-type strategies) are often decisive [4,9]. Polyfunctional isocyanates (e.g., HDI trimer/isocyanurate, biuret, polymeric MDI) increase crosslink density, improving chemical/solvent resistance and creep, but excessive crosslinking reduces compliance—problematic for large-strain electrodes (e.g., Si)—so crosslinking must be tuned to avoid brittleness and cracking [10,22].

For rechargeable-battery applications, aliphatic diisocyanates generally exhibit greater oxidative and electrochemical stability than aromatic types, making them more suitable for use as high-voltage cathode binders or in PU-based solid/gel electrolytes designed for long-term cycling and thermal exposure [6,13]. Aromatic systems provide fast cure and strong cohesion, yet are less stable under high potential, where oxidation, gas generation, and resistive interphases may form. As such, it is preferable to minimize direct contact of aromatics with oxidative or high-voltage environments or apply additional shielding strategies. Across chemistries, appropriate use of polyisocyanates can help limit swelling and maintain electrode architecture; however, over-crosslinking should be avoided to prevent brittleness and cracking, especially in electrodes with large volume changes (e.g., silicon anodes) [18,19,23]. Table 2 summarizes various isocyanates and their key features used in polyurethane/urea preparation.

#### 2.1.3. Chain Extenders

Chain extenders couple NCO-terminated prepolymers and define the hard-segment chemistry (urethane vs. urea) and hydrogen-bonding density, thereby governing microphase separation and the resulting modulus/Tg/adhesion in WBPU/WBPUU films [9,24]. Short diols typically yield urethane-rich hard segments that remain comparatively compliant (higher strain tolerance), whereas diamines (e.g., EDA, DETA, IPDA) raise urea content and cohesive interactions, often improving strength but risking excessive stiffness/brittleness when overused [24,25,26,27,28]. Multifunctional and ionic extenders/internal emulsifiers (e.g., DMPA/DMBA or tertiary-amine-based units) allow branching/crosslinking and water dispersibility while tuning particle/film behavior and, in battery formulations, wettability and Li^+^ transport; however, high ionic content and counterions can increase water/electrolyte uptake and potentially affect SEI/CEI chemistry [8,29,30].

For rechargeable battery use, diol-rich hard segments (urethane type) more readily accommodate electrode volume changes but may require a higher hard-segment fraction or additional crosslinking to resist creep and solvent attack under cycling [11,18]. Urea-rich networks maintain dimensional stability and strength at elevated temperature and during repeated cycles, but their excessive rigidity can limit compliance and cause mechanical failure in electrodes with large volume changes [24,31]. Accordingly, both extender content and structure should be tailored to balance flexibility, strength, and electrochemical compatibility for target battery systems.

### 2.2. Waterborne PU Dispersions

#### 2.2.1. Dispersion Types

Colloidal stability in WPU is typically achieved by embedding hydrophilic moieties (ionomeric or PEG-like segments) into the backbone/side chains, yielding anionic, cationic, nonionic, or zwitterionic WPUs; the stabilization mode governs dispersion robustness, substrate affinity, and behavior in battery-processing/electrolyte environments [9,30].

Anionic WPUs introduce carboxylate/sulfonate sites (e.g., via internal emulsifiers), neutralized prior to dispersion; they generally provide strong adhesion to polar substrates and broad formulation compatibility, while the ionic content must be balanced to limit water/electrolyte uptake [29,30]. Analogous principles extend to waterborne non-isocyanate PU (WNIPU) routes where carboxyl groups are installed via post-functionalization/anhydride-type chemistry before neutralization to form stable dispersions [32,33,34,35,36]. Anionic WPU design using DMPA-type incorporation is a representative and widely adopted approach [30].

Formulators create cationic WPUs by incorporating tertiary-amine motifs and protonating them to generate cationic centers before dispersion; they can strongly adsorb to negatively charged surfaces but require careful electrochemical compatibility considerations when batteries are targeted [9,37]. Cationic WNIPU/WPUU-type dispersions prepared through cyclic-carbonate/diamine pathways are representative examples of isocyanate-free waterborne systems [32,35]. High-solid-content cationic WPU formulations have also been demonstrated [37].

Nonionic WPUs rely on PEG (or related) segments for steric stabilization without counterions, which can improve salt tolerance but may increase plasticization/uptake if the hydrophilic fraction is excessive [38,39]. Zwitterionic WPUs embed paired cation/anion motifs (e.g., betaine-type structures), allowing hydration-layer stabilization with reduced sensitivity to ionic strength/pH and offering attractive interfacial transport/anti-fouling characteristics [40,41]. Anionic sites can aid wetting/adhesion to oxides/current collectors and may participate in Li^+^ coordination/transport, but excessive ionic content and counterion effects can increase swelling and impact interphase chemistry [42,43]. Cationic centers may strengthen adsorption to certain particle surfaces, yet oxidative stability at high potentials and strong salt–ion interactions can be limiting; use is more defensible at lower potentials or as engineered interlayers [9,37]. Nonionic/PEG-rich segments can improve Li^+^ solvation/ionic conduction, but uptake, creep, and oxidative durability must be controlled by segment balance and network design [39]. Zwitterionic motifs can support ion-permeable, highly hydrated interphases and reduce some ion-exchange issues, but swelling risks and limited long-term electrochemical datasets remain key gaps [44,45].

#### 2.2.2. Dispersion Routes

Stable WPU/WPUU dispersions are typically prepared by four routes—prepolymer mixing (emulsification), acetone, hot-melt, and ketimine/ketazine—and the route largely determines the solids–viscosity–particle-size window, residuals (solvent/byproducts), and film-formation quality that ultimately matter for scalable electrode coating [4,9,46].

(1) Prepolymer mixing (emulsification):

An ionomeric NCO-terminated prepolymer is dispersed into water (often under conditions minimizing NCO–H_2_O side reactions), followed by heterogeneous chain extension; cycloaliphatic diisocyanates and internal emulsifiers are commonly used to improve process stability. Historically, NMP was used to manage viscosity but should be minimized for modern, battery-facing manufacturing [4,47]. Particle size and uptake tendencies depend strongly on ionic content (e.g., DMPA level/neutralization), so designs should balance dispersion stability vs. excessive water/electrolyte uptake [29,30].

(2) Acetone process:

Prepolymer synthesis and chain extension are carried out in homogeneous acetone, allowing tighter viscosity/PSD control; when diamines are used, ketimine-blocked diamines can moderate amine–NCO reactivity and release free amine during water inversion [8,42]. The acetone route can achieve stable dispersions at lower ionic content than prepolymer mixing and clarifies how solids content, inversion conditions, and solvent–water affinity set stability thresholds [8,48].

(3) Hot-melt dispersion:

This solvent-free approach relies on high-temperature viscosity control and can yield more branched/lower-MW products with route-specific side reactions; as a result, property control and coating-quality reproducibility can be less favorable than solution-based routes [4,9].

(4) Ketimine–ketazine process:

The prepolymer is premixed with blocked diamine (ketimine) or blocked hydrazine (ketazine), then hydrolysis during dispersion releases the active extender in situ, extending pot life and suppressing gelation—particularly useful when highly reactive prepolymers are involved—provided hydrolysis kinetics and ketone byproducts are well managed [4,9]. In practice, WPU/WPUU dispersions from these routes can be readily cast into continuous films, and multiple studies report stable dispersions at ~30–35 wt% solids under controlled drying—conditions that translate well to battery-electrode coating constraints when residuals and uptake are controlled [46,48].

#### 2.2.3. Stabilization Mechanisms

WPUUs are mainly stabilized by covalently built-in internal emulsifiers and interfacial ionic/zwitterionic functionalities that generate electrostatic and/or hydration-based repulsion, dictating particle stability, solids handling, and film performance. DMPA (and DMBA) is widely used; after neutralization (e.g., TEA), anionic sites promote self-dispersion and help control particle size and water uptake, while the ionic double layer suppresses flocculation [29,30]. Zwitterionic motifs (e.g., sulfobetaine-type) can further improve stability via strongly hydrated shells (often improving salt tolerance) but may increase hydrophilicity and electrolyte uptake if overused—important for electrode/binder durability [41,44]. Chai L. et al. highlights that interfacial chemistry, particularly through zwitterionic functionalities, enhances the stability and cycling performance of LMB, achieving a high-capacity retention of 92% after 600 cycles at 1 C (155 mA h g^−1^ to 143 mA h g^−1^) [44]. Battery-relevant consequences of ionic centers and neutralization (Li^+^ coordination, swelling, electrolyte compatibility) are discussed in Section 3.2.4 and Section 3.2.5. To provide a concise overview of stabilization chemistries, Table 3 lists representative internal emulsifiers and interfacial ionic/zwitterionic/nonionic functionalities used in WPU dispersions, together with their key effects on dispersion stability and particle/film properties.

#### 2.2.4. Particle Size and Size Distribution

Particle size/PSD governs dispersion stability, film coalescence, and rheology, and is primarily tuned by hydrophilic/ionic content (e.g., DMPA level) and formulation polarity. Increasing ionic/hydrophilic functionality generally reduces particle size and narrows PSD, while polyol structure/crystallinity and overall hydrophobicity shift the stabilization demand [29,30,49]. Backbone polarity and network formation—e.g., via NCO/OH ratio—also affect nucleation/growth and PSD [50]. Process choice can intentionally yield bimodal PSD to reach higher solids with manageable viscosity, whereas more controlled/stepwise routes often give smaller, unimodal particles but higher viscosity [46,48]. For battery slurries, controlling latex particle size/PSD helps stabilize slurry rheology and coating uniformity, indirectly shaping electrode porosity and mechanical robustness [43,47].

#### 2.2.5. Film Formation, Coalescence, and MFFT

Film formation proceeds through water evaporation → particle packing → coalescence → chain interdiffusion, followed by physical/chemical consolidation (curing). Drying below minimum film formation temperature (MFFT) can leave incomplete coalescence and weak/discontinuous films; coalescing agents can temporarily lower effective MFFT but must be balanced against residuals and electrolyte compatibility in battery electrodes [4,9,30]. Post-coating crosslinking (where used) locks in cohesion/solvent resistance, but excessive crosslink density can penalize compliance—critical for volume-changing electrodes [18,19,22].

## 3. Design Strategies and Formulation Parameters

### 3.1. Fundamental Design Principles

Segmented PUs can balance ion transport and mechanical strength because their structure is built from two parts that play different roles; soft segments act as a flexible, polar medium that helps Li^+^ dissolve and move (via segmental motion), whereas hard segments form rigid, hydrogen-bonded domains that reinforce the matrix and maintain dimensional stability [9,10,13]. Because these two segments preferentially gather into different regions, PUs naturally develop microphase-separated morphologies; soft-segment–rich regions can connect to create continuous ion-conduction pathways, while hard domains serve as a mechanical scaffold that resists deformation. This “pathway + scaffold” architecture is the key reason PUs can target both conductivity and strength at the same time [13,51]. When formulated as WPU/WPUU dispersions, the platform becomes even more practical for batteries; water-based processing reduces solvent burden, and the latex/particle state offers additional parameters (particle size/PSD, coalescence, drying profile) to tune film morphology. It also simplifies the incorporation of functional additives or nanofillers that further tune coupled electrochemical–mechanical performance [6,13,51].

### 3.2. Compositional Parameters

#### 3.2.1. Soft/Hard Segment Ratio and Type Selection

In PU electrolytes, the soft segment mainly controls Li^+^ transport, while the hard segment mainly controls mechanical strength. Tuning the ratio and chemistry of these two phases is therefore the most direct way to balance conductivity vs. robustness [13,51]. For the soft segment, chemistry determines the “conductivity–stability” trade-off. PEG or PTMG typically give the highest ionic conductivity because they have low Tg and abundant ether oxygens for Li^+^ coordination; PEG-based WPU electrolytes have reported conductivities up to ~7.3 × 10^−4^ S cm^−1^ at 60 °C under tuned conditions [16,51,52]. In contrast, PCDL-based polyols generally offer better oxidative/high-voltage stability (reported operation up to ~4.5 V vs. Li/Li^+^) but often show lower conductivity (typically 10^−5^–10^−6^ S cm^−1^ at RT) because their higher Tg suppresses segmental motion [16,53]. Polyester polyols often fall in between—moderate polarity and film formation with intermediate ionic transport—making them useful as a compromise between conductivity and stability/film-forming performance [13,16].

For the hard segment, the choice of diisocyanate/chain extender sets the reinforcement level and can either preserve or hinder ion motion. For example, more flexible hard-segment chemistries (e.g., HDI-based) tend to better retain mobility, whereas more rigid hard segments (often MDI-linked) can raise strength but risk reducing ion transport if hard-domain content/connectivity becomes excessive [54,55]. In waterborne systems, this selection also affects process practicality; MDI-based prepolymers are more moisture-sensitive, so synthesis often relies on strict moisture control and/or process choices (e.g., acetone/ prepolymer routes, blocked strategies) to avoid foaming/gelation [4,8,9].

#### 3.2.2. Urethane/Urea Balance Optimization

**Figure 1:**
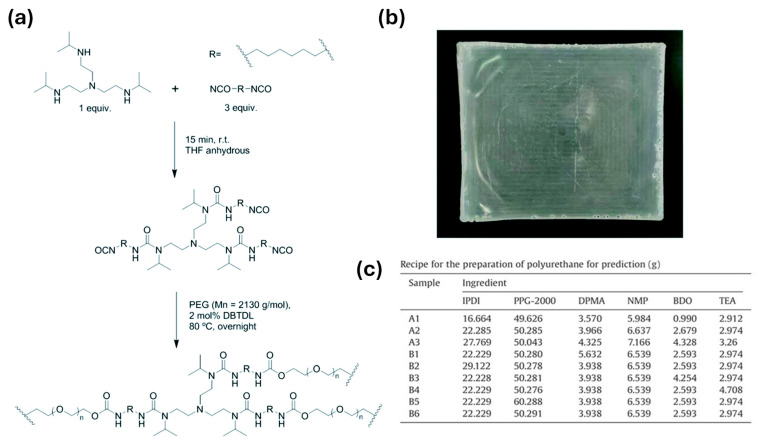
(**a**) Synthetic procedure for obtaining cross-linked aliphatic polyurethanes with dynamic crosslinking points based on HUBs. (**b**) Representative image of one of the synthesized HUB-PU networks. (**c**) Recipe for the preparation of polyurethane for prediction. Reproduced from Ref. [58] under the Creative Commons Attribution (CC BY) license © The Royal Society of Chemistry 2022.

In PU-based electrolytes, the urethane/urea ratio is one of the most important “hard-segment chemistry parameters” because it directly sets two competing outcomes: (i) hydrogen-bond density → strength/cohesion, and (ii) chain mobility → ionic transport [13,54]. Using diamine chain extenders increases urea linkages (–NH–CO–NH–), which generally form stronger and denser H-bond networks than urethane linkages. This typically improves toughness and cohesion and can also support intrinsic self-healing by strengthening reversible intermolecular interactions [24,26,56,57]. The downside is that excessive urea content can make the matrix too rigid, limiting segmental motion and thereby reducing ionic conductivity. For this reason, many designs aim for an intermediate urea fraction—enough to reinforce the polymer, but not so high that transport is strongly suppressed [13,54,58]. This balance is observed in practice; Elizalde et al. showed that urea-driven H-bonding improved mechanical robustness and self-healing in poly(urea–urethane) gel electrolytes, while overly rigid compositions led to reduced conductivity [58]. Similarly, Wang et al. reported that increasing urea-associated hard-segment content strengthened the polymer but eventually penalized ionic transport once chain mobility became too restricted [54]. Figure 1 summarizes a representative synthetic route to HUB (hindered urea bond)-allowed, dynamically crosslinked aliphatic PU networks and highlights the key building blocks used to tune urethane/urea-derived hard-domain formation.

#### 3.2.3. Chain Extender and Crosslink Density Control

**Figure 2:**
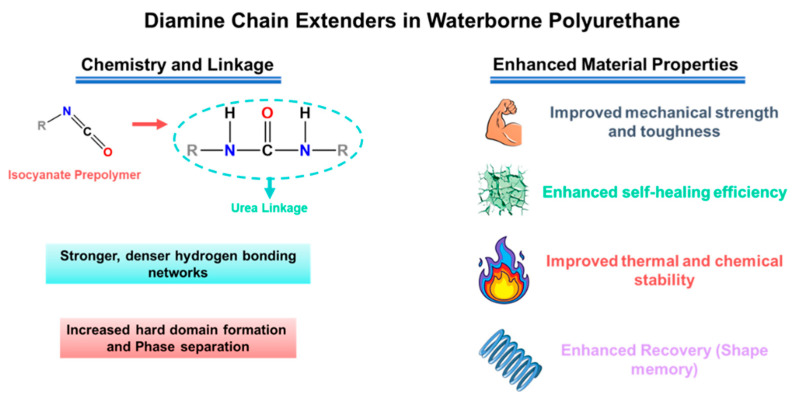
Diamine chain extenders form urea linkages in WPU/WPUU, strengthening hydrogen bonding and hard-domain formation to improve mechanical robustness and self-healing.

Chain extenders and cross-linkers are the most direct parameters for transforming the target urethane/urea ratio into the actual polymer structure, from linear chains to branched/crosslinked networks. Difunctional extenders (e.g., BDO, DEG) mainly promote linear growth and relatively uniform phase separation, which helps preserve segmental mobility (favorable for ion transport) while keeping the material flexible [24,25,26,59,60]. Multifunctional extenders/crosslinkers introduce branching points and increase network connectivity (higher “effective hard-segment connectivity”). This generally improves strength and solvent resistance, but if crosslink density is too high, chain motion becomes overly restricted and ionic transport can drop [22,61,62,63]. A practical way to increase cohesion and self-healing in WPU/WPUU is to use diamine extenders, which generate urea linkages [24,26,58]. Urea-rich hard domains typically form stronger hydrogen bonding networks than urethane-rich domains, improving toughness and allowing more effective intrinsic resilience. However, it should be noted that excessive urea/network formation can lead to excessive matrix stiffness [24,31,64]. Representative examples support these trends: sulfonate-containing diamine extenders improved healing efficiency and film robustness in WPU dispersions [65]. More broadly, polyurethane–urea elastomers leveraging strong/dynamic urea interactions have reached high strength (reported up to ~49 MPa) while maintaining high healing efficiency after thermal treatment [31]. As illustrated in Figure 2, diamine chain extenders introduce urea linkages that intensify hydrogen bonding and hard-domain cohesion, providing a direct lever to raise robustness while retaining self-healing capability.

Crosslink density is a key design lever for balancing mechanical strength and Li^+^ mobility; adding multifunctional extenders/crosslinkers (e.g., TMP) increases network connectivity and dimensional stability, but too much crosslinking can “freeze” soft-segment motion and lower ion transport [13,66]. TMP-crosslinked (PUA-based) membranes therefore often show a clear optimum at intermediate crosslinker contents (~2–5 wt%); for example, ~3 wt% TMP maintained high tensile strength while still delivering high ionic conductivity (~9.6 mS cm^−1^ at RT) and high electrolyte uptake (~245% swelling) [66]. In contrast, excessive TMP (>10 wt%) can create an overly dense network, leading to reduced swelling/uptake and a marked drop in ionic conductivity (<1 mS cm^−1^), along with increased brittleness.

#### 3.2.4. Ionic and Zwitterionic Functionalization

**Figure 3:**
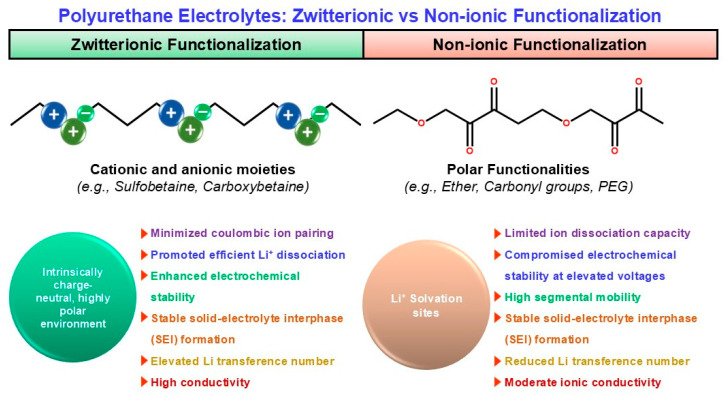
Comparative electrochemical mechanism of zwitterionic versus non-ionic polyurethane electrolytes.

In PU-based electrolytes, ionic/zwitterionic functionalization is a direct way to improve salt dissociation and Li^+^ transport; built-in charge centers reshape ion–polymer interactions and can create more continuous ion-conduction channels [13,67,68,69]. Ionic (anionic/cationic/IL-like) motifs—introduced as sulfonate/carboxylate/ionic-liquid-type pendants—can form localized ion-conducting regions and promote Li-salt dissociation. A representative ionic-liquid/imidazolium-type gel electrolyte reports σ ~ 9 × 10^−3^ S cm^−1^, t^+^ ~ 0.69, and stability up to ~4.5 V vs. Li/Li^+^, consistent with the idea that ionic functionalities help homogenize Li^+^ flux and stabilize cycling [68]. Zwitterionic motifs (e.g., sulfobetaine/carboxybetaine) provide a highly polar yet net-neutral environment that can reduce ion pairing and reduce “ion trapping,” often improving high-voltage robustness (reported >5 V) and interphase stability. Zwitterion-allowed systems (including zwitterion-assisted electrolyte designs and polyurethane-in-salt-type concepts) report conductivity on the order of 3.7 × 10^−4^ S cm^−1^ at 25 °C with favorable electrochemical stability [69,70]. By contrast, non-ionic but polar segments (ether/carbonyl; “PEG-rich” designs) mainly provide Li^+^ solvation but do not inherently drive salt dissociation or high t^+^; they can therefore show lower t^+^ and weaker oxidative stability at elevated voltages unless reinforced by complementary stabilization strategies [13,55]. Figure 3 contrasts the ion-transport picture in zwitterionic versus non-ionic polyurethane electrolytes, clarifying how fixed-charge motifs reshape the Li^+^ coordination/transport environment.

#### 3.2.5. DMPA Content and Neutralization Optimization

**Figure 4:**
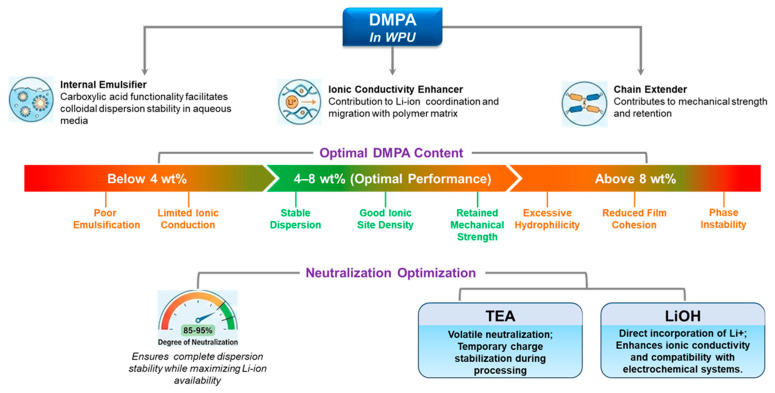
DMPA’s multifaceted role in WPU systems for battery applications.

DMPA is not only an internal emulsifier for WPUs, but also a built-in ionic site that can influence Li^+^ coordination/transport; in PEG–DMPA–IPDI single-ion WPU electrolytes, multiple studies show that DMPA units measurably affect ionic conductivity [8,9,29,30,42,71]. For battery-related formulations, DMPA content is therefore a “dual-role” lever (dispersion stability and ion-transport environment). As a practical starting window often used in WPU design, ~4–8 wt% DMPA tends to balance (i) stable dispersion formation and sufficient ionic-site density with (ii) acceptable film cohesion; too little DMPA (e.g., <~4 wt%) risks poor emulsification and limited ionic contribution, whereas too much (e.g., >~8 wt%) can over-increase hydrophilicity, swelling/uptake, and weaken cohesion [9,29,30]. Neutralization degree further sets the “active” charge density at the particle/interface and inside the film; high-but-not-complete neutralization (often ~85–95%) is commonly targeted to secure dispersion stability while avoiding unnecessary hydrophilicity [29,30]. Counterion choice also matters; TEA is a processing-friendly, volatile neutralizer, whereas LiOH directly introduces Li^+^ counterions, which can be beneficial for electrochemical compatibility and Li^+^-relevant transport motifs [29,42,71]. Figure 4 provides a consolidated view of DMPA’s multiple roles—from dispersion stabilization to electrolyte-facing swelling/impedance trade-offs—making explicit why neutralization and DMPA loading must be tuned together.

### 3.3. Key Trade-Offs in WPU/PUU Battery Systems

#### 3.3.1. Molecular Weight and Architectural Control

**Figure 5:**
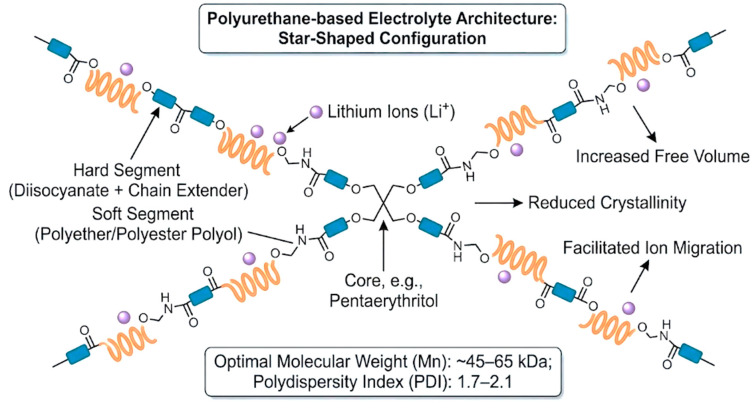
Schematic of polyurethane with multifunctional crosslinkers forming star-shaped architecture.

MWs and architecture are high-level design variables that jointly set mechanical strength, ion mobility, and processability by controlling chain entanglement, microphase separation, and segmental dynamics [13,39,72]. As a practical guideline, many studies present an intermediate Mn window (~45–65 kDa) as a workable compromise; lower Mn (<45 kDa) can improve ionic transport via higher chain mobility but may weaken tensile strength and dimensional stability, whereas higher Mn (>65 kDa) strengthens the matrix through entanglement but can slow ion motion and reduce conductivity [13,39]. A moderate dispersity Đ_M_ ≈ 1.7–2.1 is also commonly targeted for more predictable processing and consistent film formation [39]. Beyond Mn control, branched/star/hyperbranched topologies provide an additional lever to “reshape” the morphology; increasing branching can reduce crystallinity and increase amorphous/free-volume character, which can support faster ion migration without fully sacrificing mechanical stability [72,73]. These structures are typically introduced using multifunctional polyols (e.g., 3- or 4-arm polyols) and/or multifunctional crosslinkers during synthesis [21,22,61]. Hyperbranched/star-like motifs are also discussed as allowing crosslink-density and nano-/microphase tuning, helping maintain robust frameworks while preserving enough free volume for transport, and offering rheology/processability tuning important for waterborne dispersion handling and coating/electrode fabrication [21,22,46,47,48]. To visualize how architectural control reshapes the transport–mechanics landscape, Figure 5 schematically shows a star-shaped PU architecture formed by multifunctional crosslinkers.

Non-waterborne “topology-engineered” polymer electrolytes offer a helpful analogy for WPU/WPUU design. For example, hyperbranched PEO (A_2_ + B_3_ routes) and star-shaped polycarbonate/polyether architectures can suppress crystallinity and increase amorphous/free-volume character, which supports ion motion (reported up to ~6 × 10^−5^ S cm^−1^ at room temperature) while maintaining reasonable processability [51,72,73,74]. Although these systems are not WPUDs, they convey a transferable message: introducing branching or star-like topology can “loosen” the matrix, reduce crystallinity, and promote connected ion-transport pathways. In WPU/WPUU, similar effects can be implemented by using 3–4-arm polyether/polyester polyols and tri-/tetra-functional crosslinkers (e.g., TMP or pentaerythritol derivatives) [22,61,66]. When crosslink density and domain organization are properly controlled, these designs can improve toughness/elongation and solvent resistance without eliminating the free volume needed for Li^+^ migration [61,72,73].

#### 3.3.2. Ionic Conductivity vs. Storage Modulus

A persistent challenge in polymer electrolytes is the σ–E′ trade-off: high ionic conductivity typically requires high segmental mobility, whereas a high storage modulus relies on chain rigidity and strong intermolecular interactions—so improving one often harms the other [51,63]. Recent PU-based designs addressed this conflict by decoupling “mechanical reinforcement” from “ion-transport pathways.” For example, SLIC-type concepts use orthogonal H-bonding domains to reinforce the matrix while keeping ion-conducting regions active, achieving high toughness (~29.3 MJ m^−3^) with σ ~1.2 × 10^−4^ S cm^−1^ [62]. Likewise, combining dynamic covalent bonds (e.g., disulfides) with reversible H-bond motifs can allow self-healing and toughness (~12.5 MJ m^−3^) while maintaining σ ~3.2 × 10^−4^ S cm^−1^ [75,76].

A complementary route is zwitterionic functionalization. Sulfobetaine/carboxybetaine motifs provide a highly polar yet net-neutral environment that can reduce ion pairing (supporting σ) while also forming reversible ionic associations that act like dynamic crosslinks (supporting E′) [44,55,77]. In zwitterionic WPUDs studied mainly for antifouling/membrane coatings, sulfobetaine segments are reported to enrich near particle/film surfaces and generate robust hydration layers—features that, when translated to battery architectures, could help stabilize polar interfacial layers and dissipate stress without permanently blocking ion-transport pathways [40,41].

#### 3.3.3. Lithium Transference Number

The lithium transference number, t^+^, quantifies how much of the current is carried by Li^+^. Most conventional polymer electrolytes show low t^+^ (~0.1–0.3), which promotes concentration polarization and limits rate capability, so increasing t^+^ is a key design target [51,78]. A direct approach is single-ion conduction, where anions are immobilized on the polymer (e.g., carboxylate/sulfonate/phosphate sites). DMPA-based systems have reported t^+^ ~0.6–0.7, typically with some penalty in total conductivity [42,76]. Highly selective polyelectrolyte complexes (rigid-rod polymers + ionic liquids) can push t^+^ > 0.7 but often suffer from brittleness and poor processability [78,79]. Zwitterionic strategies offer a compromise: balanced cation/anion motifs can improve salt dissociation while keeping moderate selectivity, giving t^+^ ~0.4–0.5 with good conductivity [44,55,77]. Delocalized anionic motifs (e.g., sulfonyl-imide-type zwitterions) further weaken Li^+^–anion binding and can increase selectivity (e.g., t^+^ ≈ 0.43 with ~0.44 mS cm^−1^ reported) [77]. A third route is coordination/solvation engineering; increasing well-placed Lewis-base sites (ether oxygens, carbonyls) and tuning Li^+^ solvation structures can bias transport toward Li^+^, often giving t^+^ ~0.5–0.6, and in some specialized designs approaching unity, though usually with trade-offs in σ or formulation latitude [13,51].

#### 3.3.4. Interfacial Adhesion vs. Wetting

For solid(-like) polymer electrolytes and binder-allowed electrodes, adhesion and wetting must be tuned together; insufficient wetting/contact raises interfacial resistance and accelerates fade, while excessive wetting/plasticization can trigger electrolyte redistribution and mechanical instability [80,81]. A common lever is polarity/ionic functionality. Adding –OH/–COOH (or related ionic sites) can improve both adhesion to electrode surfaces and wetting, but overly high polar/ionic content can weaken mechanical strength and reduce ionic selectivity; therefore, ionic group concentration is typically tuned rather than maximized (often ~0.5–2.0 meq g^−1^ as a practical window) [80,81]. Counterion and salt choice further shift interfacial behavior; Li-salt chemistry (e.g., LiFSI vs. LiTFSI) changes wetting/adhesion via different interactions with polar groups, and neutralization chemistry during polymer synthesis (e.g., TEA vs. LiOH) can alter interfacial properties and Li^+^-transport motifs simultaneously [80,81]. Beyond bulk composition, interfacial engineering can deliver large gains without sacrificing bulk electrolyte properties. Examples include primer/intermediate adhesion layers (reported adhesion ~4.5 MPa with ~180 Ω·cm^2^ interfacial resistance), dynamic/self-healing interfacial bonding (up to ~8.7 MPa with ~42 Ω·cm^2^), and interpenetrating networks that create intimate contact (contact angle ~15°) with high adhesion (~6.2 MPa), collectively lowering interfacial impedance [80,81,82,83].

## 4. Application-Specific Design and Reference Recipes

### 4.1. Electrode Binders

#### 4.1.1. Role of Electrode Binders and Transition to Aqueous Systems

LIBs are widely used in portable electronics, electric vehicles, and grid-scale energy storage owing to their high energy density and long cycle life [84]. An electrode is typically composed of active material, conductive additives, and a small fraction of polymer binder that mechanically integrates the composite and maintains electrical contact between particles and the current collector during repeated lithiation/delithiation [11,85]. Historically, poly(vinylidene fluoride) (PVDF) dissolved in N-methyl-2-pyrrolidone (NMP) is the dominant binder system for commercial LIB electrodes. While PVDF offers good electrochemical stability and processability, the use of NMP presents several challenges: it is toxic, high-boiling, costly to recover, and requires strict environmental and safety controls [11,43]. These limitations have motivated an intensive search for aqueous binder systems, in which water replaces NMP as a processing solvent [47]. Aqueous binders can be broadly divided into water-soluble polymers (e.g., poly(acrylic acid) (PAA), carboxymethyl cellulose (CMC), sodium alginate (SA)) and waterborne latexes or dispersions (e.g., styrene–butadiene rubber (SBR), polytetrafluoroethylene (PTFE) dispersions, WPU) [11,43,47]. Beyond eliminating NMP, these systems can provide additional functionalities such as improved adhesion, elasticity, ionic transport, and self-healing [43,86].

In this section, we first survey conventional aqueous binders used in LIB and SIB electrodes (Section 4.1.3). We then cover aqueous binders, including WPU as a versatile platform for next-generation binders (Section 4.1.4), discuss key design parameters that govern their structure–property relationships (Section 4.1.5), and finally summarize application-oriented binder selection guidelines for typical graphite/Si and LFP/NMC (LiFePO_4_/LiNiMnCoO_2_) electrodes (Section 4.1.6).

#### 4.1.2. Processing Window—Drying, Residual Water, and Coatability

WPUU binders allow aqueous slurry processing (NMP-free) while maintaining dispersion and adhesion of active/conductive phases but require careful drying to avoid residual water effects [13,65]. Their segmented PU/PUU architecture supports strong cohesion/adhesion and mechanical compliance, allowing flexible and durable electrodes [5]. Reactive groups can allow in situ/post crosslinking to improve cohesion and electrolyte resistance—useful for Si anodes—yet over-crosslinking risks brittleness and cracking [18,19,23]. Representative demonstrations include interconnected multifunctional WPU binders for robust Si anodes, elastic stress-dissipative WPU binders, and WPU/CMC-Na network-type binders showing improved mechanical property and cycling stability [18,19,23,87].

#### 4.1.3. Conventional Aqueous Binders for LIB and SIB Electrodes

Conventional aqueous binders must (i) disperse active/conductive solids in water, (ii) adhere to particles and current collectors, (iii) accommodate volume changes, and (iv) remain stable within the electrode potential window [2,43,47].

PAA (poly(acrylic acid)) is a carboxyl-rich, water-soluble polyelectrolyte that strongly interacts with hydroxylated/oxide surfaces (H-bonding/ionic interactions), giving high adhesion—especially useful for Si-rich anodes [2,3,43]. Neutralization (e.g., LiOH/NaOH/NH_4_OH) is often used to tune slurry viscosity/ionization/drying [2,43]. PAA can also be applied to graphite/LFP and even high-voltage cathodes (interfacial stabilization/metal-dissolution mitigation), depending on formulation [43,88]. In SIBs, Na-PAA can improve cycling by providing strong ionic bonding and helping Na^+^ transport [2,89]. SBR and CMC/SBR hybrids are widely used in industry; CMC provides good viscosity control and adhesion to polar particle surfaces, while SBR supplies elasticity/crack tolerance [2,43,90,91]. Performance depends on SBR crosslinking and styrene/butadiene ratio (more styrene → higher modulus/Tg; more butadiene → higher elasticity) [43,92]. CMC (carboxymethyl cellulose) is low-cost and film-forming; it bonds well to graphite/Si via –OH/–COO^−^ groups and can help maintain electrode structural stability (often paired with an elastomer for high-Si electrodes) [2,43,90,93]. PTFE dispersions form a robust (often fibrillated) particle network during drying/working and can fit “dry/semi-dry” processing but usually need blending to compensate for limited intrinsic adhesion and may require energy-intensive processing [94,95]. SA (sodium alginate) is bio-based and anionic; it can form physically and ionically crosslinked networks that are attractive for high-volume-change anodes, with advanced variants (e.g., sulfonated alginate, SA@Borax-type self-healing) improving durability [86,96]. HNBR offers elastomeric compliance plus improved thermal and oxidative stability (via hydrogenation) and higher polarity (–CN) for adhesion; crosslinking can further reinforce high-energy electrodes, but cost and process-compatible crosslink chemistry matter [97,98]. For comparison, Table 4 summarizes representative aqueous binders, highlighting their key features, primary electrode applications, and common drawbacks in LIB/SIB processing and cycling.

#### 4.1.4. Waterborne Polyurethane as Advanced Electrode Binder

WPU binders are segmented polymers dispersed as colloidal particles in water. Compared with conventional aqueous binders, WPU offers a modular platform where mechanical strength/cohesion, elasticity, ionic transport, and interfacial polarity/adhesion can be tuned more independently, allowing water-based electrode processing with added functionality [43,99,100]. Representative examples show how WPU design translates into performance across electrodes [18,19,87,100,101].

(1) Si anode (WPU/CMC hybrid)

**Figure 6:**
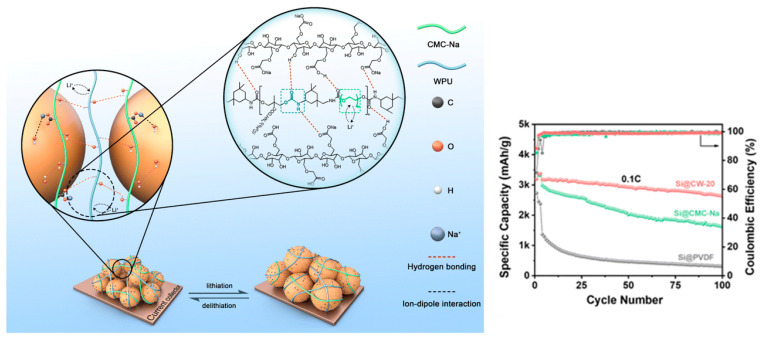
Schematic representation of the water-soluble binder network withstanding volume change and depiction of interactions among CMC-Na, WPU, and Si. Reproduced with permission from Ref. [23]. Copyright © 2024 the Royal Society of Chemistry.

A PEG-based anionic WPU blended with CMC-Na (CW-20, 20 wt% WPU) forms a flexible H-bonded network that accommodates Si volume change and improves cycling stability [23]. For Si anodes, Figure 6 depicts a water-soluble hybrid binder network (CMC-Na/WPU) designed to accommodate volume expansion while maintaining interfacial interactions with Si particles.

(2) Li–S cathode (ion-conductive 3D network)

**Figure 7:**
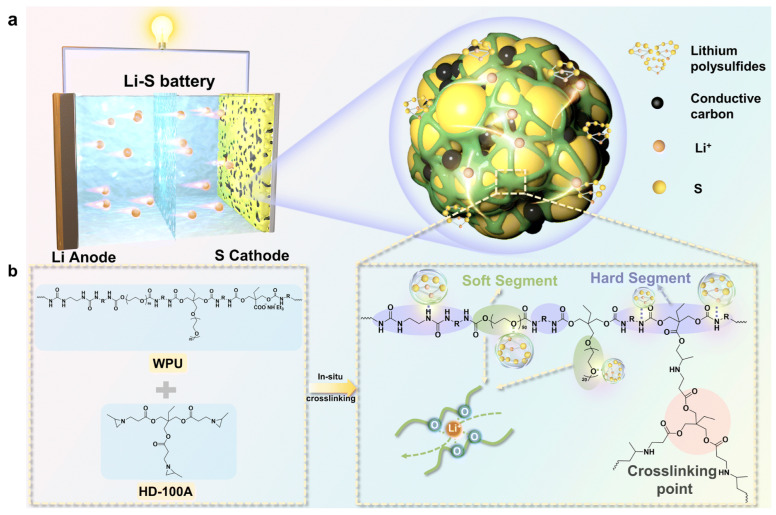
The schematic illustration of the 3D crosslinked binder acting on a Li–S battery. (**a**) The 3D network structure of CWPU encasing sulfur and carbon. (**b**) The synthesis process of the in situ crosslinked CWPU polymer. Reproduced with permission from Ref. [87]. Copyright © 2024 The Royal Society of Chemistry.

A PEG/Ymer N120-based WPU with ionic/polar groups builds an interweaving 3D ion-conductive binder network, improving rate capability and long-term cycling under practical conditions [87]. Figure 7 illustrates an in situ crosslinked CWPU 3D network that encapsulates sulfur/carbon, highlighting how WPU chemistry can be extended from “binder” to “confinement/interphase” functions in Li–S cells.

(3) Si anode (elastic stress-dissipative WPU)

**Figure 8:**
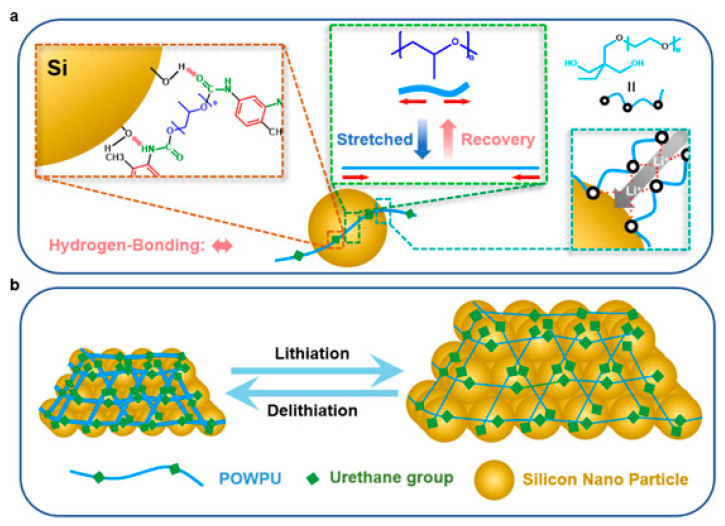
Schematic illustration of the POWPU binder: (**a**) anchoring with Si active material, helped Li^+^ transport, and stretched-recovery behavior and (**b**) buffering the pulverization of the electrode materials. Reproduced with permission from Ref. [19]. Copyright © 2024 American Chemical Society.

A nonionic PEO/PPO-based WPU (POWPU) provides elastic buffering, suppresses pulverization, and improves capacity retention versus conventional binders [19]. As summarized in Figure 8, the POWPU binder integrates anchoring motifs with helped Li^+^ transport and elastic recovery, collectively buffering electrode pulverization during cycling.

(4) Li–S cathode (multifunctional composite binder)

An anionic WPU combined with PAA and graphene (WPU–PAA–GN) strengthens adhesion and mechanical resilience while supporting electronic/ionic pathways, improving sulfur utilization and cycling [101,102].

(5) LFP cathode (soft-segment tuning)

Nonionic WPUs tailored by soft-segment choice (e.g., PTMG/PCDL/PNA) adjust film flexibility/elasticity and deliver stable cycling performance [103,104,105].

#### 4.1.5. Structure–Property Design Parameters of WPU Binders

(1) Soft-segment chemistry

**Figure 9:**
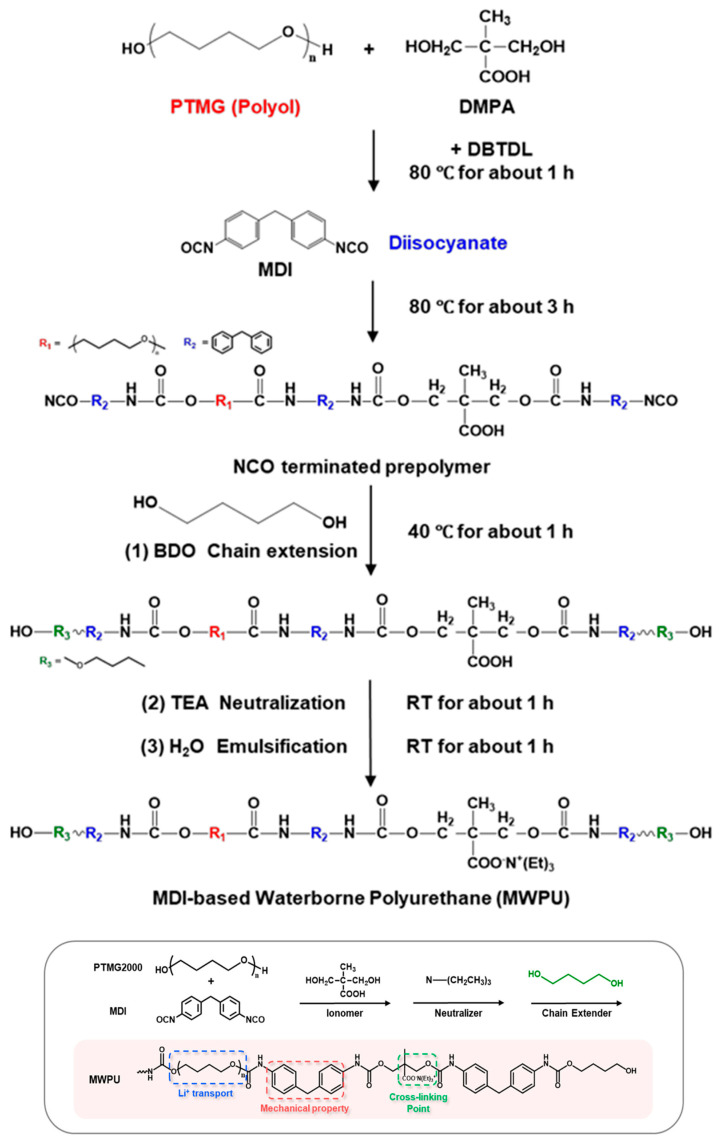
Synthesis process of MWPU and schematic of the chemical structure of the synthesized MDI-based WPU. Reproduced with permission from Ref. [18]. Copyright © 2024 American Chemical Society.

Soft-segment selection is the primary design factor in WPU binders because it simultaneously sets (i) chain mobility and stress accommodation, (ii) Li^+^ solvation/polarity (thus ion transport), and (iii) emulsion viscosity and final film mechanics [9,10]. PEO/PEG-type soft segments (ether-rich) are commonly used to increase hydrophilicity and water dispersibility, and provide abundant ether oxygens for Li^+^ coordination, which can support higher ion transport and “softer” stress relaxation [14,51,52]. Crystallinity (depending on MW and content) can add stiffness and dimensional stability but may also complicate low-temperature flexibility and swelling control [12,51]. Carbonate groups strengthen inter-segment interactions (incl. hydrogen bonding) and often yield films that remain robust yet elastic—useful when long-term mechanical stability is prioritized. The trade-off is that higher polarity and rigidity can reduce segmental mobility unless balanced with more flexible ether-rich components [15,16]. Low-Tg, flexible ether (PTMG-type) soft segments are frequently used to improve elasticity and accommodate repeated expansion and contraction (e.g., in high-capacity anodes). Their molecular weight strongly affects dispersion rheology and film tensile response, so MW/content must be tuned together with hard-segment content [14,106]. A practical takeaway from comparative WPU studies is that polyether-rich soft segments often give lower emulsion viscosity and higher toughness than polyester-rich analogs, which can translate into easier coating and more crack-tolerant electrode films (when other variables are held comparable) [10,12]. Figure 9 provides the synthesis workflow and the resulting chemical structure of an MDI-based WPU (MWPU), serving as a structural reference point for discussing ionic-group/neutralization-driven property shifts [18].

(2) Molecular weight and topology control

The NCO:OH stoichiometric ratio is a primary handle to set chain length, branching/crosslink tendency, and residual functionality in WPU networks. Near-stoichiometric ratios typically favor mostly linear or lightly branched chains, which improves flexibility and coatability but can limit cohesion if the network is too “loose.” Large deviations can leave unreacted NCO/OH (or drive uncontrolled side reactions), leading to defective networks, poorer mechanical strength, and less predictable processing, so battery binders usually require a practical optimum that balances flexibility with peel strength and cycling robustness [10,17,50]. Beyond stoichiometry, MW and dispersity both improve mechanical robustness and slurry/latex handling; higher Mn increases entanglement (better cohesion) but raises viscosity, while overly broad Đ can cause non-uniform flow and film formation [10,29]. Chain extension and crosslinking (via diols/diamines or multifunctional agents) provides additional control to raise cohesion and solvent resistance—but must be moderated so that mobility and processability are not over-penalized [22,24,25,26]. For electrode binders, mild-to-moderate 3D networking is often beneficial because it improves swelling resistance and interfacial durability under cycling. A representative example is a WPU for Si anodes where controlled stoichiometry plus post-crosslinking chemistry produced a more cohesive network and improved cycling stability [18,19,107].

(3) Ionic groups and neutralization

WPU binders typically rely on built-in ionic groups to stabilize latex particles in water and to tune wetting/adhesion on electrode surfaces. Common motifs include carboxylates (e.g., via DMPA), sulfonates (e.g., sulfonated polyols), and cationic ammonium sites (via protonated tertiary amines); these determine particle charge, dispersion stability, and surface energy [29,30,37,42,65]. Zwitterionic groups (e.g., sulfobetaine/carboxybetaine/phosphobetaine) embed both positive and negative charges in the same unit and can be placed in either hard or soft segments. They are known to create strongly hydrated interphases (often linked to antifouling) and, compared with purely ionic systems, can improve barrier/coating durability by reducing ion migration-driven defects; mixed ionic designs (e.g., carboxylate + sulfonate) can also broaden the pH range for stable dispersions [30]. Neutralization chemistry and counterion choice (Li^+^, Na^+^, TEA^+^) strongly affect water uptake, microstructure, and electrochemical response. Li^+^-neutralized carboxylate/sulfonate sites can form more compact ionic clusters and are often discussed as beneficial in Li-salt environments (supporting Li^+^-compatible pathways), whereas Na^+^ typically gives higher hydration/swelling and can aid film formation [29,30,69,108,109]. TEA^+^ often boosts dispersion stability and wetting via bulky, weakly coordinating cations, but excessive TEA^+^ can increase impedance or degrade barrier performance [29,30]. Overall, more ionic content improves wetting and dispersion stability, but too much can drive excessive water/electrolyte uptake, high slurry viscosity, drying defects, and poorer EIS/barrier behavior; zwitterionic motifs are sometimes used to soften this trade-off by keeping strong hydration while suppressing uncontrolled ion transport in the cured film [29,30,40,41]. Finally, particle size and size distribution (PSD) also matter for coatability at high solids; smaller particles (e.g., D50 ~ 50–70 nm) can give smoother films but higher viscosity, whereas larger particles reduce viscosity at the cost of uniformity. A bimodal/broad PSD can allow higher solids (reported >60 wt%) at manageable viscosity by improving packing efficiency [46,48].

(4) Dynamic/supramolecular networks (CAN/UPy) for micro-crack healing

A clear way to improve the durability of WPU binders is to introduce dynamic covalent and/or supramolecular motifs (e.g., CANs and UPy) so the network can rearrange under mild stimuli and close microcracks. Disulfide-based WPUs often heal via bond exchange and chain diffusion, with activation energies typically in the ~20–55 kJ mol^−1^ range; for example, Ea ≈ 20.4 kJ mol^−1^ (effective healing above ~65 °C) and Ea ≈ 36.1 kJ mol^−1^ with ~94% repair efficiency in dual-dynamic system [75,110,111]. UPy provides strong but reversible quadruple H-bond crosslinks, allowing repeatable healing (and sometimes recyclability) while retaining cohesion [75,112,113]. Combining CAN chemistry + UPy supramolecular bonding is therefore a practical route to simultaneously target high strength and repeatable microdamage repair in WPU films/binders [75,111,112,113]. For battery use, these dynamic/supramolecular architectures are mainly valued because they can suppress crack growth during cycling and extend electrode service life, but their long-term stability under harsh electrochemical conditions (e.g., high-voltage cathodes, concentrated carbonate electrolytes, aqueous environments) still needs more direct validation; other dynamic motifs (DA, imine/Schiff-base, metal–ligand, etc.) should be benchmarked under realistic electrode–electrolyte pairs [111,114].

#### 4.1.6. Application-Oriented Binder Selection Guidelines for Graphite/Si and LFP/NMC Electrodes

Based on Section 4.1.2, Section 4.1.3 and Section 4.1.4, Table 5 lists application-specific aqueous-binder “recipes” by electrode chemistry because no single binder architecture is optimal across all electrodes and operating windows [2,11,43,47,115]. For Si-rich anodes, formulations that combine high elasticity with controlled cohesion/crosslinking (e.g., WPU-based designs) are repeatedly used to better tolerate large volume swings and maintain particle/current-collector contact under cycling [3,18,19,43,116]. For LFP (and by extension high-voltage cathodes such as NMC), binder choice must also emphasize interfacial chemistry (wetting/adhesion/impedance stability), so cathode-specific recipes—including ionic/zwitterionic design—are often more effective than “one-size-fits-all” aqueous binders [45,47,103,105].

### 4.2. GPE/SPE Matrices and Li-Metal Interlayers

#### 4.2.1. Design Principles

Polymer electrolytes for LMBs can be GPEs (polymer + trapped liquid) or SPEs (ions move mainly through amorphous polymer segments) [117,118,119,120,121]. In both, the polymer provides shape/mechanical stability, while the ion-conducting phase provides Li^+^ transport [117,119,120,121]. For long-life Li-metal cells, the Li–metal interface must be engineered in addition to the bulk matrix [80,117]. A soft, deformable “cushion/buffer” interlayer can redistribute local mechanical stress during Li plating/stripping, reducing stress concentration at surface asperities and thereby lowering the likelihood of dendrite nucleation [80,122,123]. Single-ion (anion-immobilizing) interlayers and adhesive interphases help maintain intimate contact and homogenize Li^+^ flux [80,123,124]. Practically, three coupled variables dominate design: (i) ionic conductivity (σ) set by segmental dynamics and Tg, (ii) the σ–E′ trade-off (stiffening typically reduces mobility), and (iii) Li^+^ transference number (t^+^) (low t^+^ accelerates concentration polarization) [39,51,78,117,124]. Accordingly, “winning” designs usually (i) keep the conducting phase amorphous/mobile, (ii) add separate reinforcing motifs/phases rather than globally hardening the matrix, and (iii) raise t^+^ using single-ion or zwitterionic and anion-trapping concepts [44,51,62,69,124].

#### 4.2.2. Gel Polymer Electrolytes

GPEs are polymer networks swollen with liquid electrolyte (salt + carbonate solvents/plasticizers), made either by swelling a pre-formed polymer or by in situ polymerization in the presence of liquid electrolyte [66,68,117,125]. Their main advantage is that the liquid-rich phase dominates ion transport, so room-temperature conductivity is typically ~10^−4^–10^−3^ S cm^−1^, while the polymer skeleton provides form stability and good electrode contact (often lowering interfacial resistance vs. dry SPEs) [68,117]. Key limitations are also liquid-derived; volatility/leakage, phase separation, and reduced thermal safety, and over-plasticization can weaken the matrix, so it becomes less effective at mechanically resisting dendrite growth [68,80]. Thus, GPE design is essentially a balance between solvation/transport and mechanical/thermal robustness, often addressed by tuning matrix chemistry + crosslink density and using less volatile and safer plasticizers [68,126,127].

#### 4.2.3. Solid Polymer Electrolytes

SPEs are near solvent-free systems where Li salts are dissolved and solvated directly by a solid polymer host, allowing robust films, intimate electrode contact, and scalable processing with reduced flammability and no leakage compared with liquid/GPE systems [119,120,121,126,128]. Li^+^ transport in SPEs is mainly governed by the amorphous phase above Tg. Segmental motion creates free volume and coordination-site hopping pathways, so effective hosts typically combine (i) high salt-dissociating polarity and (ii) sufficient chain mobility while remaining electronically insulating [120,121,126,129,130]. PEO-based SPEs are classic examples because ether oxygens coordinate Li^+^, but room-temperature conductivity is often limited (~10^−7^–10^−3^ S cm^−1^) by crystallinity; additional constraints include a narrower oxidative stability window (~4.0 V), low t^+^ (~0.2–0.3), and modest modulus that can allow dendrite penetration under demanding conditions [51,78,120,124,131]. Carbonate-based SPEs are an important alternative for higher-voltage operation; carbonate functionalities (e.g., PVCA-type or related copolymers) can improve oxidative stability (~4.5–5.0 V), and in situ polymerization can reduce interfacial resistance; typical room-temperature conductivities (~10^−5^–10^−4^ S cm^−1^) are often higher than crystalline PEO under comparable conditions [16,53].

#### 4.2.4. WPU-Based Electrolytes

(1) Baseline WPU electrolytes

WPU electrolytes are attractive for Li batteries because their segmented hard/soft morphology can combine mechanical toughness and adhesion (hard domains) with ion transport (soft segments), while allowing water-based processing [13,14]. A key design parameter is to use ether- and/or carbonyl-rich soft segments to increase Li^+^ coordination/dissociation and improve σ and sometimes t^+^—but carbonyl binding must be balanced to avoid over-trapping Li^+^ [13,16]. In practice, reported WPU-based SPE/GPE conductivities commonly fall in the ~10^−5^–10^−3^ S cm^−1^ range at room temperature, and can be further boosted in composite/plasticized designs; carbonate-type PU SPEs can reach t^+^ ~0.6 in well-designed systems [6,52,85]. A widely used performance strategy is WPU/PEO blending; WPU helps suppress PEO crystallinity and strengthens the film/interface, while PEO supplies dense ether sites for Li^+^ hopping—often giving ~10^−4^ S cm^−1^ at room temperature with improved flexibility/stability versus neat PEO [52,132]. Representative examples you already cite can be stated compactly as: (i) WPU/PEO blend SPE with improved conductivity and stable cycling [132]; (ii) plasticized WPU gel electrolyte tuned by LiCF_3_SO_3_–PC and PEG/DMPA (σ up to ~10^−2^ S cm^−1^ at 25 °C) [133]; (iii) WPU single-ion design with immobilized anions to raise selectivity (higher t^+^) [42,54,134].

(2) Reinforcement with filler/micelle

**Figure 10:**
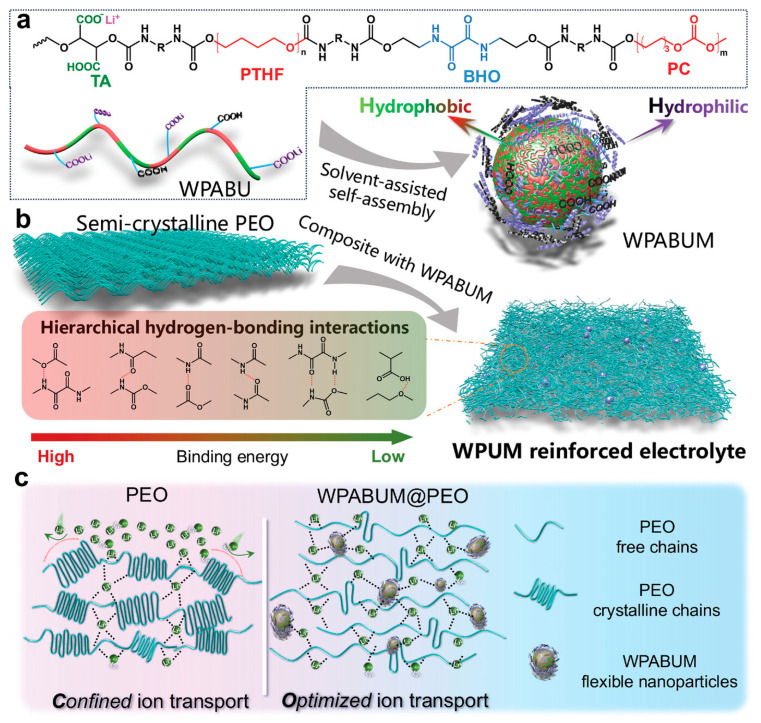
(**a**) Chemical structure and schematic representation of WPABU. (**b**) Schematic representation of the preparation of WPABUM@PEO. (**c**) Schematic illustration of WPABUM@PEO for tuned Li^+^ transport. Reproduced with permission from Ref. [135]. Copyright © 2024 Wiley-VCH GmbH.

WPU micelles can act as “soft” multifunctional nanofillers in PEO/Li-salt matrices; they add urethane/urea-rich interaction sites (H-bonding/ionic interactions), suppress PEO crystallinity, and stabilize a more amorphous, conductive phase [135]. In a representative case (WPUM@PEO), this translated to ~800 h stable Li‖Li cycling at 0.1 mA cm^−2^ (EO:Li = 8:1) with low polarization. Figure 10 outlines the chemical design of WPABU and its hybridization with PEO (WPABUM@PEO), emphasizing the intended continuous Li^+^ transport pathway while preserving polymer mechanical integrity. Inorganic/reactive additives provide a parallel “hard-filler” reinforcement route; adding a small amount of nano-Al_2_O_3_ (Lewis-acidic filler) plus LiOH (active modifier) into PU/LiTFSI composites is used to tune nanostructure and ion transport [85,136]. Similar LiOH/Al_2_O_3_ synergy is also seen outside WPU, where alumina can increase amorphous fraction and provide high-energy interfaces for Li^+^ hopping, while LiOH supplies Li^+^ and modifies polymer –OH groups, improving conductivity vs. LiOH-only systems [136]. An alternative composite route mixes an aqueous PEO–LiTFSI phase into a PU organic phase; multilevel H-bonding plus flexible micelle deformation yields strong mechanical stability, with ~800 h (Li‖Li) stability at 0.1 mA cm^−2^ and >400 cycles (Li‖LFP) at 1C reported [135].

(3) Hard-segment content and crosslink density to balance σ–E′

WPU electrolyte design typically faces a σ–E′ trade-off: increasing hard-segment content and/or crosslink density raises modulus and dimensional stability, but it usually reduces ionic conductivity because Li^+^ transport mainly proceeds through the soft, segmentally mobile domains [6,13,39,62]. Andersson et al. showed that keeping the hard-segment fraction below ~35% (soft segment ≥ ~65%) can preserve a mechanically stable network while maintaining sufficient soft-phase connectivity for LiTFSI transport; higher hard-segment contents produced stiffer, more glassy matrices with suppressed segmental dynamics and lower σ [6]. Crosslinking similarly strengthens the electrolyte, but an intermediate crosslinking level can be optimal; moderate crosslinking may improve dimensional stability without fully blocking segmental motion, whereas excessive crosslinking restricts Li^+^ hopping [66,117]. Jin et al. reported an optimum dual-crosslinking condition (TMP/PEG ≈ 0.08), achieving high room-temperature conductivity (~9.6 mS cm^−1^) together with improved stability, while under- or over-crosslinking degraded either mechanics or ion transport [66].

(4) Single-ion functionalization for high t^+^

Single-ion (ionically functionalized) WPU electrolytes immobilize the anion on the polymer backbone so that Li^+^ becomes the dominant mobile charge carrier, which can raise t^+^ and reduce concentration polarization in LMB [79,124]. In conventional salt-in-WPU designs (e.g., LiTFSI in carbonyl/ether-rich soft segments), both Li^+^ and the anion migrate, and t^+^ commonly remains modest (≈0.2–0.4), allowing strong concentration gradients to develop under current [52,78,137]. In WPU systems, single-ion behavior is typically implemented in three ways: (i) ionic diols/chain extenders (sulfonate/carboxylate/phosphonate/TFSI-like) incorporated during polymerization—often into hard segments to lock the anion while adding structural stability, (ii) ionically functional polyols that place fixed anions along the soft segments so the anion is immobilized directly in the Li^+^-transport domain, and (iii) post-functionalization of pre-formed WPU chains with sulfonate or TFSI-like groups [42,54,124,134].

Mechanistically, low-t^+^ electrolytes can develop Li^+^ depletion near the Li surface during plating, which amplifies local fields and promotes non-uniform Li deposition and dendrite growth; high-t^+^ (single-ion or anion-trapping) designs flatten interfacial concentration/potential profiles and promote more uniform Li^+^ flux, delaying polarization-driven failure [78,79,124].

A representative non-WPU example (PEG-based SIPE with a perfluorosulfonate anion) illustrates the potential benefit of high-t^+^ single-ion architectures, showing t^+^ near unity and long dendrite-free Li‖Li cycling, motivating analogous WPU/WPUU single-ion designs for solid-state lithium-ion batteries (SLIBs) or LMBs [52,69,73,79].

#### 4.2.5. Composite/Hybrid Electrolytes

Nanofillers such as LLZO, LATP, Mxene, and SiO_2_ are widely used in GPE/SPE matrices because they can raise ionic conductivity, reinforce the polymer, and stabilize the Li interface. LLZO nanofibers can form percolated Li^+^ pathways, improving room-temperature conductivity and oxidative stability while helping block dendrite growth [138,139,140,141]. Vertically aligned LATP can further bias Li^+^ transport directionally and outperform isotropic composites [142]. SiO_2_-based (including Li-salt-modified) fillers can increase matrix rigidity and interfacial cohesion and improve resistance to dendrite penetration [140,143]. A key determinant of performance is interfacial compatibility/interaction (IC) between the polymer and filler surface [142]. IC is typically mediated by H-bonding or specific interactions between polar groups in the polymer (e.g., urethane/urea) and surface –OH/phosphate groups on ceramics, which improves dispersion, reduces interfacial resistance, and supports a more stable Li interphase during cycling [143]. In this context, segmented PU/WPU chemistries provide an additional lever to balance σ vs. mechanical reinforcement in hybrid matrices [6,143]. Practically, matrix design focuses on selecting filler type/morphology/loading and ensuring homogeneous dispersion (often ~10–20 wt% filler in common polymer hosts), while Li-metal contact layers often use crosslinked, polar-group-rich PU frameworks (sometimes with ceramic fillers) to buffer volume change and suppress dendrite growth [143,144]. Representative WPU electrolyte examples support processability and performance. Cheng et al. prepared a WPU film by casting/drying and then introduced LiCF_3_SO_3_–PC, showing conductivity increases and charge-transfer resistance decreases with plasticizer content; the 70 wt% LiCF_3_SO_3_–PC formulation reached ~10^−3^ S cm^−1^ at 25 °C [145]. Cong et al. reported an organic-solvent-free PEG-based WPU SPE with LiTFSI, where the tuned composition delivered 7.3 × 10^−4^ S cm^−1^ at 60 °C and >4.8 V stability, allowing stable cycling in an LFP/SPE/Li cell [52].

**Figure 11:**
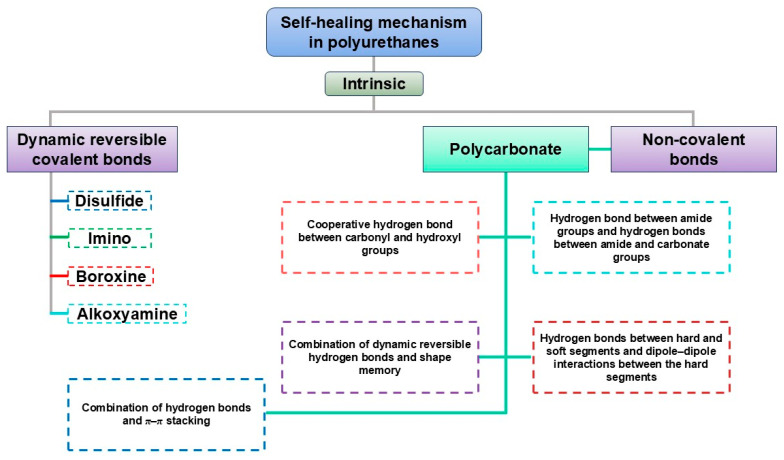
Overview of intrinsic self-healing mechanism in PUs. These networks additionally regulate SEI/CEI formation: ion-coordinating groups such as ether oxygens, carbonyls, and sulfonates alter Li^+^ solvation and promote the development of LiF-rich or inorganic-dominant interphases that are mechanically robust and more resistant to dendrite propagation. Their dynamic character further accommodates interfacial volume changes without delamination [76,82,110,114,127].

Finally, dynamic covalent and supramolecular networks in PU/PUU-based GPE/SPEs help maintain Li contact by allowing stress dissipation and microcrack/void repair, which can smooth the Li interface and slow dendrite growth [76,82,86]. These designs combine reversible H-bond networks and/or exchangeable covalent bonds (e.g., disulfide, imine/Schiff base, acylhydrazone, boronic ester, Diels–Alder) and can be extended with coordination/host–guest/π–π interactions to add recoverable cohesion and ion-coordination sites [75,110,114]. Self-healing architectures can also be paired with high-t^+^ concepts (including single-ion designs) to reduce concentration polarization and promote more uniform Li deposition [76]. Overall, the “mechanics + reversibility” combination provides high modulus at small strain to resist penetration, while bond exchange and chain diffusion relax stress and continuously restore interfacial contact during cycling [76,86]. Pei F. et al. [82]. show that the solid-state polymer electrolyte, with dynamic disulfide and hydrogen bonds, provides self-healing and stable interfacial contact, enabling over 6000 h of cycling in Li||Li cells and 700 cycles at 0.3 C in Lithium–Sulfur batteries. To position battery-oriented designs within broader PU chemistry, Figure 11 overviews intrinsic self-healing mechanisms in PUs, which can be repurposed to stabilize electrode–electrolyte contacts under cycling.

Dual-dynamic WPU/WPUU electrolytes can be designed by combining multiple H-bond motifs (e.g., urea/thiourea/amide-assisted associations) with dynamic covalent units (e.g., disulfide, imine/Schiff base, acylhydrazone), introduced either in the backbone or via chain extenders [75,82,110,114]. This strategy increases toughness while allowing (near) room-temperature healing through bond exchange and supramolecular rearrangement [75,82,110]. Ion transport is mainly supported by ether-rich or zwitterionic soft segments, whereas hard-segment fraction and supramolecular cross-links are adjusted to retain the modulus needed for dendrite resistance [6,44,69]. In Li-metal interlayers, thin PU/WPU-based supramolecular coatings—often combined with ceramic nanofillers or lithiophilic functionalities—can further homogenize Li^+^ flux and buffer deformation, improving dendrite suppression through coupled mechanical/chemical stabilization [141,144,146]. Pei et al. demonstrated a poly(ether-urethane) SPE with intrinsic self-healing that can be cast directly onto electrodes to build an integrated electrode–electrolyte interface [82]. Disulfide exchange plus urethane H-bond rearrangement restored interfacial continuity and delivered mechanically robust SPEs with high ionic conductivity, elevated t^+^, and a wide stability window; Li|PTMG–HDI–BHDS|Li cells cycled stably for >6000 h, and the strengthened interface supported stable cycling in SPAN- and S@CB-based cathodes [82].

Recent improvements in interfacial design, such as molecular polarity tuning and coordination modulation, have aimed to stabilize interfaces and optimize transport mechanisms. In order to maximize interfacial chemistry and prevent interfacial degradation, these methods usually depend on exact molecule or ion-level design. Luo, J et al. [147] integrated a polymer gel electrolyte within fiber structures through in situ polymerization, optimizing both ionic conductivity and mechanical compliance for wearable power sources. Wearable electronics applications can benefit from this design’s strong electrochemical performance and stable interfaces. With an energy density of 128 Wh kg^−1^, the better polymer gel electrolyte employed in fiber lithium batteries increases their scalability and makes them viable for a variety of applications, including space exploration, firefighting, and human–computer interfaces, even in challenging conditions.

In contrast, the current study shows that WPUs with segmented polymer architecture and intrinsically programmable polar functional groups may efficiently control interfacial conditions. The cooperative interactions between urethane/urea moieties and lithium species contribute to enhanced interfacial stability and controlled lithium-ion transport, without the need for complex coordination engineering or additional functional additives. While sharing the common principle of interface-level regulation, this polymer-based strategy offers a more versatile and environmentally benign pathway for interfacial stabilization, highlighting the broader applicability of waterborne polymer design in both LIB and LMB battery systems beyond the specific materials studied here.

#### 4.2.6. WPU-Based Li-Metal Interlayers for Homogenizing Ion Flux

**Figure 12:**
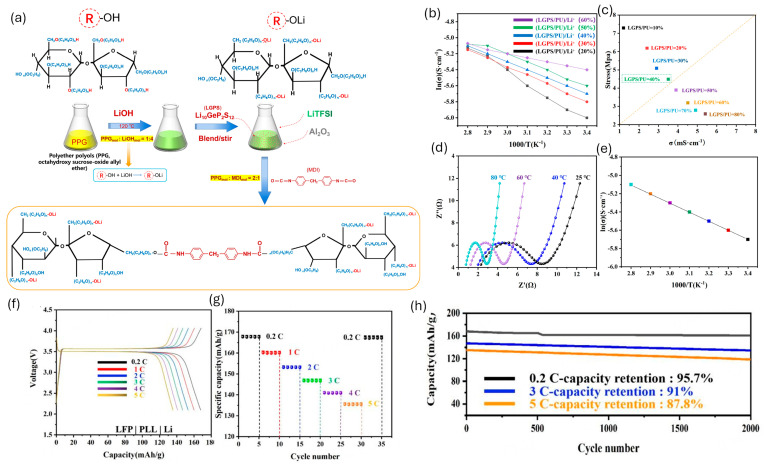
(**a**) Preparation process of PLL [(PU-LGPS)/Li^+^]; (**b**) Arrhenius plots for the ionic conductivities of different proportions of (LGPS/PUx)/Li^+^; (**c**) Relationship between ionic conductivity and stress, PLL at different temperatures; (**d**) AC impedance; (**e**) Arrhenius plots for the ionic conductivities (40%); (**f**) LFP|PLL|Li, the first charge/discharge curves for 0.2 C, 1 C, 2 C, 3 C,4 C, and 5 C at ambient temperatures; (**g**) Capacity with various C-rates; and (**h**) Long-cycling properties offered based on varying C-rates. Reproduced from Ref. [146] under the Creative Commons Attribution (CC BY) license © OAE Publishing Inc. 2023.

WPU interlayers can act as artificial-SEI-like coatings on Li metal; their segmented, hydrogen-bonded urethane/urea matrices coordinate Li^+^, provide viscoelastic buffering, and help level ion flux and maintain intimate contact during plating/stripping, which collectively reduces dendrite-prone interfacial instability [6,144,148]. Key design knobs are ether-rich soft segments (Li^+^ solvation/transport), ionic or single-ion functionalities (higher t^+^), and crosslink density/hard-segment tuning to balance compliance (stress dissipation) with dendrite resistance under electrochemical stress [6,76]. Representative examples include a hyperbranched PU electrolyte incorporating IL/LiTFSI that improves salt dissociation and forms microphase-separated ion pathways while strengthening electrode/electrolyte contact [74]. A PU/LGPS (Li_10_GeP_2_S_12_) composite electrolyte (LGPS dispersed in PPG-rich soft domains) reaches t^+^ ≈ 0.56 with σ ≈ 3.1 × 10^−3^ S cm^−1^ (25 °C) and allows long-life LFP full-cell cycling (reported up to 2000 cycles) [146]. Figure 12 summarizes the preparation of a PU–LGPS composite electrolyte and its coupled transport–mechanics metrics (Arrhenius conductivity and conductivity–stress relationships), underscoring the practical σ–E′ co-optimization challenge.

### 4.3. Separator Coatings and Primers

High-ceramic separator coatings are often designed as binder-in-ceramic structures, where a small amount of polymer forms a continuous phase around ceramic particles; this increases puncture resistance and reduces thermal shrinkage, improving separator safety under mechanical deformation or thermal abuse [149,150,151,152,153]. WPUU is attractive for these coatings because it combines mechanical strength, thermal stability, intrinsic hydrophilicity, and compatibility with water-based processing [151,154,155]. In practice, WPUU (i) keeps coatings crack-free even at ultra-high ceramic loadings (80–90 wt%), (ii) improves electrolyte wettability and cycling stability, and (iii) reinforces puncture resistance through polymer-network toughening with ceramic reinforcement [151,152]. Coating/primer durability is largely governed by crosslink density, which is commonly tuned by the NCO:OH ratio (typically ~1.1–1.6) and post-curing that consumes residual NCO groups [83,154]. Crosslinking reduces solubility/swelling in liquid electrolyte and improves heat/dimensional stability (>150 °C), allowing strong suppression of PE/PP shrinkage (e.g., >95% dimensional retention after 160 °C exposure for post-cured PUU layers) [149,150,151]. Zhang et al. also showed that TMP/GLY/TEOA crosslinking (optimal ~1.5% TEOA) yields tough, highly extensible WPUU films (44.20 MPa tensile strength; 596.61% elongation), supporting their use as separator primers/coatings [156,157].

Liu et al. tuned the soft-segment fraction in WPU to control crystallinity, microphase separation, and crosslink density, achieving high strength/toughness together with very strong adhesion, which supports WPU’s suitability for high-performance primer/adhesive layers [158]. Patent disclosures further support dual-layer primer concepts: a first, more crosslinked NCO/OH-based primer layer provides strong adhesion and mechanical resilience, while a second, less crosslinked (or uncrosslinked) layer is used to improve interfacial contact and ion transport; blocked (or partially blocked) isocyanates allow delayed, post-application crosslink activation [83]. Primer selection depends on separator substrate chemistry. For PE/PP, which are hydrophobic and chemically inert, practical approaches rely on a polar/hydrophilic interlayer (often aided by plasma/corona pretreatment) to improve wetting and anchoring; WPUU or acrylic dispersions bearing polar groups (e.g., –OH, –COO^−^) improve coating uniformity and durability [151,152]. Bio-derived ANF coatings on cationized PP also improve mechanical robustness and thermal stability, translating to better rate capability and cycling performance [159]. For PI/aramid, primers typically use more reactive/thermosetting chemistries (e.g., crosslinkable PUUs or imidizable systems) to secure stronger interfacial bonding; aramid–zirconia coated separators are a representative example of this route [153]. For cellulose-based CNF/BC membranes, primers help ensure compatibility with ceramic coatings; sulfonated CNF coatings can support ionic/thermal performance and ceramic attachment, and polar WPUUs can further reinforce interfacial adhesion and composite structural stability [160]. Overall, effective separator/electrode priming combines (i) a polar interlayer for wetting/anchoring with (ii) controlled crosslinking for durability, while avoiding over-crosslinking that could hinder interfacial ion access [83,151].

## 5. Summary and Outlook

WPU/WPUU platforms lie at the intersection of manufacturing sustainability through water-based processing and mechanics–interface engineering enabled by segmented morphology and tunable adhesion. Their broader adoption will depend on managing moisture and extractables, ensuring electrolyte compatibility, and addressing the intrinsic transport–mechanics trade-off. A central barrier is that battery electrolytes and interphases are highly sensitive to trace water and low-molecular-weight residues. Despite advantages in processing, WPU/WPUU face key limitations from hydrophilic/ionic groups that drive water/electrolyte uptake, causing swelling, plasticization, and inferior long-term mechanical/chemical resistance versus solvent-borne PU. Persistent trade-offs exist between conductivity/modulus in electrolytes and adhesion/swelling in binders, with inadequate characterization of high-voltage stability, cycling, moisture, in-electrolyte mechanics, and impedance hindering progress. Future studies should therefore treat drying protocol, residual-moisture quantification, and extractables control as mandatory variables rather than peripheral details. Practical progress will likely come from low-hygroscopic stabilization strategies and counterion design, coupled with process-aware post-treatments, so that waterborne advantages are retained without compromising interphase quality. In this context, internal emulsifiers and neutralization chemistry should be viewed not only as dispersion enablers but also as determinants of the Li^+^ coordination environment, swelling, and interfacial chemistry. A clear opportunity is counterion engineering and ionic-center selection that reduce hygroscopicity while maintaining coatability and adhesion in electrodes.

At the materials-design level, polymer electrolytes can benefit from two complementary concepts: immobilizing anions (single-ion designs) and forming soft, continuous domains that stabilize ion solvation. These approaches can improve the cation transference number while preserving ionic conductivity. A persistent bottleneck, however, is the coupling between conductivity and mechanical stiffness; raising modulus often suppresses segmental mobility and lowers ionic conductivity. Strategies that can partially ease this trade-off include (i) segmented PU morphologies that create well-connected ion pathways while keeping the matrix mechanically supportive, (ii) ceramic–polymer hybrids where WPU/WPUU improves particle dispersion/contact and adds toughness, and (iii) topology and crosslink optimization that improves dimensional stability without fully freezing the motions that allow ion transport.

For LMB, the target is not only high ionic conductivity but also stable, low-impedance contact under repeated plating/stripping. WPU/WPUU chemistries that form mechanically compliant yet reduction-tolerant interlayers—and that stabilize interphase formation—are promising, especially when evaluated under realistic current densities and stack pressures. For high-voltage cathodes and separator coatings, oxidative stability and metal-ion management become dominant, favoring application-specific WPU designs rather than a single universal formulation. Self-healing and dynamic-network strategies are also being explored to reduce microcracking, contact loss, and interfacial degradation, but their practical relevance will ultimately hinge on stable electrochemical behavior, low extractables, and sustained impedance control under full-cell conditions. Finally, the diversity of WPU chemistries will translate into impact only when studies are directly comparable—through shared benchmark conditions and reference use-cases—so that robust, application-relevant design guidelines can emerge and guide WPU/WPUU toward reliable battery technologies.

## Figures and Tables

**Table 1:** Summarizes, in a compact form, the relationship between polyol type, characteristic structure, key advantages, and features in battery applications.

Polyol Family	Key Advantages (Binder/Adhesive)	Battery Application—Advantages	Battery Application—Limitations
Polyether (PPG, PTMG, etc.)	High flexibility and elongation; good hydrolytic and humidity resistance	Good flexibility and strain tolerance; ether oxygens can contribute to Li^+^ solvation and ionic transport	Limited oxidative/electrochemical stability at high voltages; may swell in common carbonate electrolytes
Polyester	High tensile strength and cohesion; good oil and solvent resistance; strong adhesion to polar substrates	Good adhesion to oxide/phosphate active materials and metallic current collectors	Susceptible to hydrolysis and chemical attack by moisture and electrolyte degradation products
Polycarbonate diol (PCDL)	Excellent hydrolytic, thermal, and oxidative stability; very good resistance to oils and chemicals; high toughness and elastic recovery	Forming mechanically robust, hydrolytically and oxidatively stable networks; can maintain strong particle–binder interfaces	Limited segmental mobility and ionic conductivity; often requires blending with more flexible, ion-solvating polyols
Polycaprolactone diol	Good balance of strength, flexibility, and abrasion resistance	Good mechanical strength and creep resistance, suppressing cracking/delamination	Excessive crystallinity may lead to embrittlement, poor low-temperature performance
Acrylic polyol / PU–acrylic hybrid	High gloss, hardness, and block resistance; excellent weatherability and color retention; shear strength enhancement in adhesives	Improved mechanical strength, crack resistance, and dimensional stability of composite electrodes	Limited intrinsic ion-solvating ability; high Tg and rigid domains can reduce flexibility and hinder ionic transport
Fluorinated polyol	Extremely low surface energy; excellent water and oil repellency; high chemical and thermal stability	Outstanding oxidative/chemical stability; forming stable, passivating interphases and improving high-voltage tolerance	Extremely low polarity and poor ion-solvating power; tends to reduce wetting and adhesion to active materials and current collectors
Hydrophilic polyol (PEG, ionic, etc.)	Improves prepolymer dispersibility and WPU particle stability; improves wetting of hydrophilic substrates or ionic conductivity	Improved Li^+^ solvation, ionic conductivity, and electrode wetting in polymer electrolytes	Excessive hydrophilicity promotes water and electrolyte uptake, swelling, and plasticization
Rubber-like polyols (HTNR, HTPB, etc.)	Very high elasticity and energy dissipation; excellent low-temperature flexibility; good adhesion to flexible substrates	High stretchability and crack tolerance to solid polymer electrolytes, improving durability under large volume changes	Low modulus and dimensional stability; relatively low oxidative and thermal stability
Branched polyols (3-, 4-functional, hyperbranched)	Controlled crosslink density and 3D network formation; improved cohesion, chemical resistance	Useful for designing dimensionally stable, solvent-resistant binder networks and gel polymer electrolytes	Excessive crosslinking may result in rigidity and brittleness, leading to interfacial delamination during repeated lithiation/delithiation

**Table 2:** Di- and multi-isocyanates used in polyurethane/urea preparation.

Isocyanates	Chemical Structure	Relative Reactivity	Features and Remarks
Toluene diisocyanate (TDI) isomers	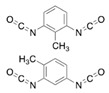	Very high; reacts rapidly with polyols/amines; 1st NCO reacts 1.4 times faster than MDI; 2nd NCO reacts 3.3 times slower than MDI	Unsuitable as a binder material for high-voltage cathodes due to poor oxidative stability and safety concerns
Methylene diphenyl diisocyanate (MDI)	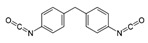	Rapid and stable reaction; crosslinks easily, promotes 3D network formation; overall curing faster than TDI	Limited oxidative stability, so mainly relevant for lower-voltage electrodes or structural adhesive layers
Hexamethylene diisocyanate (HDI)	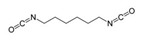	Moderate to low; aliphatic, lower reactivity than TDI/MDI; excellent UV/yellowing resistance	Good oxidative stability, attractive for WPU/WPUU binders in high-voltage cathodes
IPDI	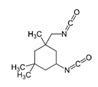	Moderate to low; comparable or lower reactivity than HDI	Good oxidative stability; good for high-voltage cathode binders and flexible SPE/GPE
H12MDI	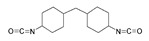	Moderate to low; comparable or lower reactivity than HDI	Good weathering and chemical resistance; good for mechanically robust, high-voltage-stable WPUU binders
Isocyanurate (trimer)	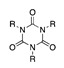	Moderate; self-catalyzed trimerization; reacts slower with polyols than monomers	Improve thermal and chemical stability of battery binders; excessive rigidity may embrittle electrodes and hinder stress relaxation during cycling
Polymeric MDI	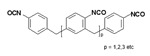 Blend of isomers and oligomers	High to very high. Reactivity can be controlled; high with catalyst, suited for foams	Less suitable for high-voltage cathode binders but potentially useful in structural or low-voltage components

**Table 3:** Internal emulsifiers and interfacial functionalities in WPU dispersions.

Component	Description	Key Effects in WPU Dispersions
Dimethylolpropionic acid (DMPA)	Anionic internal emulsifier, carboxyl groups neutralized for dispersion	Allows stable anionic dispersions, controls particle size, and influences thermal and mechanical properties
Dimethylolbutanoic acid (DMBA)	Alternative internal emulsifier with different hydrophilicity	Smaller particle sizes, less hydrophilic films
Ionic Groups (anionic/cationic)	Provide electrostatic repulsion for stability	Affects particle size, dispersion stability, drying, water absorption
Zwitterionic Groups	Contain both + and − charges, strong hydration shell	Improved dispersion stability, hydrophilicity, antifouling, biomedical applicability
Nonionic hydrophilic segments (PEG, PEO, poly(oxyalkylene))	Pendant or backbone PEO or related ether segments providing steric stabilization without ionic charge	Improved compatibility with electrolytes; increase Li^+^ solvation and ionic transport; high contents can promote water/electrolyte uptake and plasticization

**Table 4:** Summary of representative aqueous binders: composition, key features, primary applications, and limitations in LIB/SIB electrodes.

Binder	Key Features	Primary Application	Disadvantages
Sodium carboxymethyl cellulose (CMC)	A biopolymer; water-soluble, strong hydrogen bonding	Widely used for anodes, especially Si-based, due to strong adhesion	Brittle if used alone; limited elasticity
Styrene-butadiene rubber (SBR)	Excellent elasticity and flexibility	Typically used with CMC for improved mechanical properties and flexibility in anodes.	Weak adhesion to hydrophilic oxide surfaces; usually requires a polar co-binder (e.g., CMC)
Poly(acrylic acid) (PAA)	Water-soluble weak polyelectrolyte with carboxylic acid groups	Strong interaction with Si/oxide surfaces, high mechanical strength, good for Si-based and alloying anodes	Swelling/viscosity strongly depend on MW and degree of neutralization; can be too rigid or too hydrophilic
Sodium alginate (SA)	Bio-based polysaccharide; High stiffness/modulus, strong adhesion.	Strong interfacial bonding, high stiffness, suitable for Si-rich anodes, renewable/environmentally friendly	Swelling and pH sensitivity; properties require careful control or co-binders for optimal performance
Poly(tetrafluoroethylene) (PTFE)	Chemically inert fluoropolymer used as aqueous dispersion	Excellent chemical/thermal stability, allows NMP-free or dry/semi-dry processing	Poor intrinsic adhesion to inorganic particles and current collectors; requires specific processing and/or co-binders
Hydrogenated nitrile butadiene rubber (HNBR)	Partially saturated nitrile rubber with pendant nitrile groups	High mechanical strength, elasticity, and chemical/thermal resistance; improves electrode and cycling stability	Needs suitable crosslinking chemistry; higher cost than conventional binders (CMC/SBR, PVDF)

**Table 5:** Application-oriented aqueous binder recipes for graphite/Si anodes and LFP/NMC cathodes.

Electrode Type	Recommended Aqueous Binder Recipes
Graphite anodes/moderate-voltage LFP cathodes (≈3.7–3.8 V)	CMC/SBR hybrid systems (CMC + SBR latex) PAA-containing systems in partial replacement of CMC/SBR (e.g., CMC/PAA/SBR blends)
Si-rich and other high-volume-change anodes (Si, SiOx, Si-graphite blends)	PAA-rich binders (PAA alone or PAA/SBR, CMC/PAA/SBR) Alginate-based systems (SA; self-healing SA@Borax nanocomposites) WPU-hybrid binders (e.g., WPU/CMC hybrids such as CW-20, WPU-modified PAA systems) Nonionic WPU binders for Si (e.g., POWPU-type elastomeric WPUs)
High-voltage cathodes (high-Ni NMC, LNMO, etc.)	CMC/SBR systems tuned for higher modulus and adhesion PAA-containing binders (CMC/PAA/SBR) for stronger interfacial bonding and improved structural stability Alginate-based binders (SA, modified SA) for stiff, strongly bound cathode films WPU binders, both nonionic and ionic/zwitterionic types, tailored for high-voltage cathodes

## Data Availability

No new data were created or analyzed in this study.

## Abbreviations

ANF	aramid nanofiber
BC	bacterial cellulose
BDO	1,4-butanediol
CEI	cathode–electrolyte interphase
CMC	carboxymethyl cellulose
CNF	cellulose nanofiber
DEG	diethylene glycol
DMBA	dimethylolbutanoic acid
DMPA	dimethylol propionic acid
EIS	electrochemical impedance spectroscopy
GLY	glycerol
GPE	gel polymer electrolyte
HDI	hexamethylene diisocyanate
H_12_MDI	4,4′-dicyclohexylmethane diisocyanate
HNBR	hydrogenated nitrile butadiene rubber
HUB	hindered urea bond
IC	interfacial compatibility
IL	ionic liquid
IPDI	isophorone diisocyanate
LFP	lithium iron phosphate (LiFePO_4_)
LGPS	lithium germanium phosphorus sulfide
LIB	lithium-ion battery
LMB	lithium metal battery
LiFSI	lithium bis(fluorosulfonyl)imide
LiOH	lithium hydroxide
LiTFSI	lithium bis(trifluoromethanesulfonyl)imide
MDI	4,4-methylene diphenyl diisocyanate
MFFT	minimum film formation temperature
NCO	isocyanate functional group (–N=C=O)
NMC	lithium nickel manganese cobalt (LiNi_x_Mn_y_Co_z_O_2_)
NMP	N-methyl-2-pyrrolidone
PAA	poly(acrylic acid)
PC	propylene carbonate
PCDL	polycarbonate diol
PE	polyethylene
PEG	poly(ethylene glycol)
PEO	poly(ethylene oxide)
PI	polyimide
PP	polypropylene
PPG	poly(propylene glycol)
PSD	particle size distribution
PTFE	polytetrafluoroethylene
PTMG	poly(tetrahydrofuran) diol
PU	polyurethane
PUU	poly(urethane-urea)
PVDF	poly(vinylidene fluoride)
SA	sodium alginate
SBR	styrene–butadiene rubber
SEI	solid–electrolyte interphase
SLIC	supramolecular lithium-ion conductors
SLIB	solid-state lithium-ion battery
SPE	solid polymer electrolyte
TEA	triethylamine
TEOA	triethanolamine
Tg	glass transition temperature
TMP	trimethylolpropane
WNIPU	waterborne non-isocyanate PU
WPU	waterborne polyurethane (general term for waterborne polyurethane dispersions)
WPUU	waterborne polyurethane-urea (used when urea linkages are introduced via diamine chain extension)
Symbols	
Đ_M_	dispersity
E′	storage modulus
t^+^	transference number
σ	ionic conductivity

## References

[1] Shi C., Yu M. (2023). Flexible solid-state lithium-sulfur batteries based on structural designs. Energy Storage Mater..

[2] Ma Y., Ma J., Cui G. (2019). Small Things Make Big Deal: Powerful Binders of Lithium Batteries and Post-Lithium Batteries. Energy Storage Mater..

[3] Eshetu G.G., Figgemeier E. (2019). Confronting the Challenges of Next-generation Silicon Anode-based Lithium-ion Batteries: Role of Designer Electrolyte Additives and Polymeric Binders. ChemSusChem.

[4] Noble K.L. (1997). Waterborne Polyurethanes. Prog. Org. Coat..

[5] Abrahamsen G.M., Lequeux Z.A.B., Kemp L.K., Wedgeworth D.N., Rawlins J.W., Newman J.K., Morgan S.E. (2025). Morphology Control in Waterborne Polyurethane Dispersion Nanocomposites through Tailored Structure, Formulation, and Processing. Langmuir.

[6] Andersson R., Hernández G., See J., Flaim T.D., Brandell D., Mindemark J. (2022). Designing Polyurethane Solid Polymer Electrolytes for High-Temperature Lithium Metal Batteries. ACS Appl. Energy Mater..

[7] Oh S., Stache E.E. (2024). Recent advances in oxidative degradation of plastics. Chem. Soc. Rev..

[8] Sardon H., Irusta L., Fernández-Berridi M.J., Luna J., Lansalot M., Bourgeat-Lami E. (2011). Waterborne Polyurethane Dispersions Obtained by the Acetone Process: A Study of Colloidal Features. J. Appl. Polym. Sci..

[9] Honarkar H. (2018). Waterborne polyurethanes: A review. J. Dispers. Sci. Technol..

[10] Chattopadhyay D., Raju K.V.S.N. (2007). Structural engineering of polyurethane coatings for high performance applications. Prog. Polym. Sci..

[11] Cholewinski A., Si P., Uceda M., Pope M., Zhao B. (2021). Polymer Binders: Characterization and Development toward Aqueous Electrode Fabrication for Sustainability. Polymers.

[12] Diao S., Zhang Y., Zhao C., Wang M., Yu J. (2022). Preparation of Waterborne Polyurethane Based on Different Polyols: The Effect of Structure and Crystallinity. J. Polym. Res..

[13] Lv Z., Tang Y., Dong S., Zhou Q., Cui G. (2022). Polyurethane-Based Polymer Electrolytes for Lithium Batteries: Advances and Perspectives. Chem. Eng. J..

[14] Cui P., Li Y., Liu Y., Wang S., Tang X., Ye Y., Su H., Sun C. (2024). Polyether-Based Polyurethane Electrolyte for Lithium Metal Battery: A Perspective. RSC Adv..

[15] Kojio K., Furukawa M., Motokucho S., Shimada M., Sakai M. (2009). Structure−Mechanical Property Relationships for Poly (Carbonate Urethane) Elastomers with Novel Soft Segments. Macromolecules.

[16] Eriksson T., Gudla H., Manabe Y., Yoneda T., Friesen D., Zhang C., Inokuma Y., Brandell D., Mindemark J. (2022). Carbonyl-Containing Solid Polymer Electrolyte Host Materials: Conduction and Coordination in Polyketone, Polyester, and Polycarbonate Systems. Macromolecules.

[17] Joseph Vincent B., Natarajan B. (2014). Waterborne Polyurethane from Polycaprolactone and Tetramethylxylene Diisocyanate: Synthesis by Varying NCO/OH Ratio and Its Characterization as Wood Coatings. Open J. Org. Polym. Mater..

[18] Lim E.Y., Kim J.-O., Lee E., Kwon T., Bin Park J., Ko J.-W., Cho K.Y., Lee J.H. (2024). Interconnected Multifunctional Waterborne Polyurethane Binder for Structural Robustness of Si Anodes in Lithium-Ion Batteries. Ind. Eng. Chem. Res..

[19] Kim J.O., Kim E., Lim E.Y., Kwon T., Kim I.J., Lee J., Ko J.W., Lee J.H. (2024). Stress-Dissipative Elastic Waterborne Polyurethane Binders for Silicon Anodes with High Structural Integrity in Lithium-Ion Batteries. ACS Appl. Energy Mater..

[20] Athawale V.D., Nimbalkar R.V. (2011). Waterborne Coatings Based on Renewable Oil Resources: An Overview. J. Am. Oil Chem. Soc..

[21] Chen H., Sun X., Qing N., Tang L. (2025). Synthesis and Properties of Branched Waterborne Polyurethane Thickener with Multiple Arms. Polym. Eng. Sci..

[22] Tramontano V.J., Blank W.J. (1995). Crosslinking of waterborne polyurethane dispersions. J. Coat. Technol..

[23] Sun X., Lin X., Wen Y., Dong F., Guo L., Song Z., Yang Z., Liu H., Li X., Xu X. (2024). A water-soluble binder with high adhesive strength for stable Zn–I_2_ battery based on I_3_^−^ anchored in a porous carbon host. Green Chem..

[24] Delpech M.C., Coutinho F.M.B. (2000). Waterborne Anionic Polyurethanes and Poly(Urethane-Urea)s: Influence of the Chain Extender on Mechanical and Adhesive Properties. Polym. Test..

[25] Yang Z., Cui X. (2022). Effect of Chain Extenders with Different Functionalities on the Properties of Single-Component Waterborne Polyurethane Ink Binders. RSC Adv..

[26] Santamaria-Echart A., Ugarte L., Gonzalez K., Martin L., Irusta L., Corcuera M.A., Eceiza A. (2016). Synthesis of waterborne polyurethane-urea dispersions with chain extension step in homogeneous and heterogeneous media. J. Colloid Interface Sci..

[27] Jhon Y.K., Cheong I.W., Kim J.H. (2001). Chain extension study of aqueous polyurethane dispersions. Colloids Surf. A.

[28] Jang J.S., Jhon Y.K., Cheong I.W., Kim J.H. (2002). Effect of process variables on molecular weight and mechanical properties of water-based polyurethane dispersion. Colloids Surf. A.

[29] Nanda A.K., Wicks D.A. (2006). The influence of the ionic concentration, concentration of the polymer, degree of neutralization and chain extension on aqueous polyurethane dispersions. Polymer.

[30] Pandya H., Mahanwar P. (2020). Fundamental Insight into Anionic Aqueous Polyurethane Dispersions. Adv. Ind. Eng. Polym. Res..

[31] Hao Y., Zhu G., Li B. (2024). Self-Healing Polyurethane-Urea Elastomers with High Strength and Toughness Based on Dynamic Hindered Urea Bonds and Hydrogen Bonds. Ind. Eng. Chem. Res..

[32] Wu Z., Dai J., Tang L., Qu J. (2019). Sorbitol-Based Aqueous Cyclic Carbonate Dispersion for Waterborne Nonisocyanate Polyurethane Coatings via an Environment-Friendly Route. J. Coat. Technol. Res..

[33] Gomez-Lopez A., Panchireddy S., Grignard B., Calvo I., Jerome C., Detrembleur C., Sardon H. (2021). Poly(Hydroxyurethane) Adhesives and Coatings: State-of-the-Art and Future Directions. ACS Sustain. Chem. Eng..

[34] Ma S., Zhang H., Sablong R.J., Koning C.E., van Benthem R.A.T.M. (2018). T-Butyl-Oxycarbonylated Diamines as Building Blocks for Isocyanate-Free Polyurethane/Urea Dispersions and Coatings. Macromol. Rapid Commun..

[35] Ma S., van Heeswijk E.P.A., Noordover B.A.J., Sablong R.J., van Benthem R.A.T.M., Koning C.E. (2018). Isocyanate-Free Approach to Water-Borne Polyurea Dispersions and Coatings. ChemSusChem.

[36] Zhang W., Wang T., Zheng Z., Quirino R.L., Xie F., Li Y., Zhang C. (2023). Plant Oil-Based Non-Isocyanate Waterborne Poly(Hydroxyl Urethane)s. Chem. Eng. J..

[37] Li M., Liu F., Li Y., Qiang X. (2017). Synthesis of stable cationic waterborne polyurethane with a high solid content: Insight from simulation to experiment. RSC Adv..

[38] Yin Y., Feng M., Yao J., Niu J. (2024). Synthesis and Characterization of Nonionic Waterborne Polyurethane and Application to Wool Fabric Finishing. J. Wuhan Univ. Technol.-Mater. Sci. Ed..

[39] Bocharova V., Sokolov A.P. (2020). Perspectives for Polymer Electrolytes: A View from Fundamentals of Ionic Conductivity. Macromolecules.

[40] Wang C., Ma C., Mu C., Lin W. (2014). A Novel Approach for Synthesis of Zwitterionic Polyurethane Coating with Protein Resistance. Langmuir.

[41] Mao H., Zhang Q., Lin L., He X., Wang L. (2023). A Self-Healable and Recyclable Zwitterionic Polyurethane Based on Dynamic Ionic Interactions. Polymers.

[42] Wen T.-C., Wang Y.-J., Cheng T.-T., Yang C.-H. (1999). The effect of DMPA units on ionic conductivity of PEG–DMPA–IPDI waterborne polyurethane as single-ion electrolytes. Polymer.

[43] Chen B., Zhang Z., Xiao M., Wang S., Huang S., Han D., Meng Y. (2024). Polymeric Binders Used in Lithium Ion Batteries: Actualities, Strategies and Trends. ChemElectroChem.

[44] Chai L., Zou Z., Yang Z., Yang G. (2024). The Role of Zwitterionic Crosslinks in Facilitating Ion Conduction, Lithium Deposition, and Stable Interface Formation for Polymer Electrolyte-Based Lithium Metal Batteries. J. Mater. Chem. A Mater..

[45] Jin B., Dolocan A., Liu C., Cui Z., Manthiram A. (2024). Regulating Anode-Electrolyte Interphasial Reactions by Zwitterionic Binder Chemistry in Lithium-Ion Batteries with High-Nickel Layered Oxide Cathodes and Silicon-Graphite Anodes. Angew. Chem. Int. Ed..

[46] Peng S.J., Jin Y., Cheng X.F., Sun T.B., Qi R., Fan B.Z. (2015). A New Method to Synthesize High Solid Content Waterborne Polyurethanes by Strict Control of Bimodal Particle Size Distribution. Prog. Org. Coat..

[47] Rolandi A.C., de Meatza I., Casado N., Forsyth M., Mecerreyes D., Pozo-Gonzalo C. (2024). Unlocking Sustainable Power: Advances in Aqueous Processing and Water-Soluble Binders for NMC Cathodes in High-Voltage Li-Ion Batteries. RSC Sustain..

[48] Peng S., Jin Y., Sun T., Qi R., Fan B., Cheng X. (2014). Synthesis of High Solid Content Waterborne Polyurethanes with Controllable Bimodal Particle Size Distribution. J. Appl. Polym. Sci..

[49] Quane E.J., Elders N., Newman A.S., van Mourik S., Williams N.S.J., van den Berg K.J., Ryan A.J., Mykhaylyk O.O. (2024). Synthesis, Morphology, and Particle Size Control of Acidic Aqueous Polyurethane Dispersions. Macromolecules.

[50] Xian W., He M., Zheng R., Liu Z., Lu M., Chu Y., Cao H., Lu Z. (2025). The Effect of NCO/OH Ratio on the Synthesis and Behavior of Solvent-Free CO_2_-Based Waterborne Polyurethane. J. Coat. Tech. Res..

[51] Mindemark J., Lacey M.J., Bowden T., Brandell D. (2018). Beyond PEO—Alternative Host Materials for Li^+^-Conducting Solid Polymer Electrolytes. Prog. Polym. Sci..

[52] Cong B., Song Y., Ren N., Xie G., Tao C., Huang Y., Xu G., Bao J. (2018). Polyethylene Glycol-Based Waterborne Polyurethane as Solid Polymer Electrolyte for All-Solid-State Lithium Ion Batteries. Mater. Des..

[53] Xu J., Hu Y., Zhang M., Cao J., Wang M., Hong B., Lai Y. (2024). High-Performance Copolymerized Polycarbonate-Based Solid Electrolytes for Lithium Metal Batteries. ACS Appl. Energy Mater..

[54] Wang N., Chen X., Sun Q., Song Y. (2022). Single-Ion Conducting Polymer Electrolytes Based on Random Polyurethane–Urea with Different Diisocyanate Structures for Lithium Batteries. ACS Appl. Energy Mater..

[55] Wen T.-C., Luo S.-S., Yang C.-H. (2000). Ionic Conductivity of Polymer Electrolytes Derived from Various Diisocyanate-Based Waterborne Polyurethanes. Polymer.

[56] Han K.R., Saddique A., Lyu J., Kim J.C., Cheong I.W. (2024). Microphase Separation Effects on Surface Scratch-Healing and Thermo-Mechanical Properties of Self-Healing Copolymers with Dynamic Covalent Bonds. ACS Appl. Polym. Mater..

[57] Park J.I., Choe A., Kim M.P., Ko H., Lee T.H., Noh S.M., Kim J.C., Cheong I.W. (2018). Water-adaptive and repeatable self-healing polymers bearing bulky urea bonds. Polym. Chem..

[58] Elizalde F., Amici J., Trano S., Vozzolo G., Aguirresarobe R., Versaci D., Bodoardo S., Mecerreyes D., Sardon H., Bella F. (2022). Self-healable dynamic poly(urea-urethane) gel electrolyte for lithium batteries. J. Mater. Chem. A.

[59] Li F., Liang Z., Li Y., Wu Z., Yi Z. (2022). Synthesis of Waterborne Polyurethane by Inserting Polydimethylsiloxane and Constructing Dual Crosslinking for Obtaining the Superior Performance of Waterborne Coatings. Compos. B Eng..

[60] Lei L., Zhong L., Lin X., Li Y., Xia Z. (2014). Synthesis and Characterization of Waterborne Polyurethane Dispersions with Different Chain Extenders for Potential Application in Waterborne Ink. Chem. Eng. J..

[61] Iezzi E.B., Daniels G.C., Sutyak K., Camerino E. (2024). Impact of Cross-Linker Structure on the Properties of Durable and Selectively Degradable Silyl-Containing Polyurethane Networks. ACS Appl. Polym. Mater..

[62] Mackanic D.G., Yan X., Zhang Q., Matsuhisa N., Yu Z., Jiang Y., Manika T., Lopez J., Yan H., Liu K. (2019). Decoupling of Mechanical Properties and Ionic Conductivity in Supramolecular Lithium Ion Conductors. Nat. Commun..

[63] Lu G., Zhang Y., Zhang J., Du X., Lv Z., Du J., Zhao Z., Tang Y., Zhao J., Cui G. (2023). Trade-Offs between Ion-Conducting and Mechanical Properties: The Case of Polyacrylate Electrolytes. Carbon Energy.

[64] Morávková Z., Podešva J., Shabikova V., Abbrent S., Dušková-Smrčková M. (2025). Hydrogen Bonding of Trialkyl-Substituted Urea in Organic Environment. Molecules.

[65] Xiao Y., Huang H., Peng X. (2017). Synthesis of Self-Healing Waterborne Polyurethanes Containing Sulphonate Groups. RSC Adv..

[66] Jin L., Lim H., Bae W., Song S., Joo K., Jang H., Kim W. (2024). Crosslinked Gel Polymer Electrolyte from Trimethylolpropane Triglycidyl Ether by In Situ Polymerization for Lithium-Ion Batteries. Gels.

[67] Cui G., Man J., Ji M., Song X., Zhang Y., Zhang X., Li J., Li J. (2025). Ionic Response Mechanism of Lubricating Properties of Zwitterionic Polymer Brushes through Molecular Dynamics. ACS Appl. Mater. Interfaces.

[68] Zhou T., Zhao Y., Choi J.W., Coskun A. (2021). Ionic Liquid Functionalized Gel Polymer Electrolytes for Stable Lithium Metal Batteries. Angew. Chem. Int. Ed..

[69] Wang K., Koverga V., Maslekar N., Wu F., Kuphl R., Lyu X., Deshpande P., Guo H., Seol H., Degraff W. (2025). Novel Zwitterionic Polyurethane-in-Salt Electrolytes with High Ion Conductivity, Elasticity, and Adhesion for High-Performance Solid-State Lithium Metal Batteries. Adv. Energy Mater..

[70] Lu F., Gao X., Wu A., Sun N., Shi L., Zheng L. (2017). Lithium-Containing Zwitterionic Poly(Ionic Liquid)s as Polymer Electrolytes for Lithium-Ion Batteries. The J. Phys. Chem. C.

[71] Wen T.C., Wang Y.J. (1999). Application of Experimental Design to the Conductivity Optimization for Waterborne Polyurethane Electrolytes. Ind. Eng. Chem. Res..

[72] Liu Y., Zeng Q., Li Z., Chen A., Guan J., Wang H., Wang S., Zhang L. (2023). Recent Development in Topological Polymer Electrolytes for Rechargeable Lithium Batteries. Adv. Sci..

[73] Hao S.-M., Liang S., Sewell C.D., Li Z., Zhu C., Xu J., Lin Z. (2021). Lithium-Conducting Branched Polymers: New Paradigm of Solid-State Electrolytes for Batteries. Nano Lett..

[74] Wang H., Li X., Zeng Q., Li Z., Liu Y., Guan J., Jiang Y., Chen L., Cao Y., Li R. (2024). A Novel Hyperbranched Polyurethane Solid Electrolyte for Room Temperature Ultra-Long Cycling Lithium-Ion Batteries. Energy Storage Mater..

[75] Rong J., Zhong J., Yan W., Liu M., Zhang Y., Qiao Y., Fu C., Gao F., Shen L., He H. (2021). Study on Waterborne Self-Healing Polyurethane with Dual Dynamic Units of Quadruple Hydrogen Bonding and Disulfide Bonds. Polymer.

[76] Gan H., Zhang Y., Li S., Yu L., Wang J., Xue Z. (2021). Self-Healing Single-Ion Conducting Polymer Electrolyte Formed via Supramolecular Networks for Lithium Metal Batteries. ACS Appl. Energy Mater..

[77] Yan S., Lu Y., Liu F., Xia Y., Li Q., Liu K. (2023). Zwitterionic Matrix with Highly Delocalized Anionic Structure as an Efficient Lithium Ion Conductor. CCS Chem..

[78] Fong K.D., Self J., Diederichsen K.M., Wood B.M., McCloskey B.D., Persson K.A. (2019). Ion Transport and the True Transference Number in Nonaqueous Polyelectrolyte Solutions for Lithium Ion Batteries. ACS Cent. Sci..

[79] Porcarelli L., Shaplov A.S., Bella F., Nair J.R., Mecerreyes D., Gerbaldi C. (2016). Single-Ion Conducting Polymer Electrolytes for Lithium Metal Polymer Batteries That Operate at Ambient Temperature. ACS Energy Lett..

[80] Seymour I.D., Quérel E., Brugge R.H., Pesci F.M., Aguadero A. (2023). Understanding and Engineering Interfacial Adhesion in Solid-State Batteries with Metallic Anodes. ChemSusChem.

[81] Ransom B., Ramdas A., Lomeli E., Fidawi J., Sendek A., Devereaux T., Reed E.J., Schindler P. (2023). Electrolyte Coatings for High Adhesion Interfaces in Solid-State Batteries from First Principles. ACS Appl. Mater. Interfaces.

[82] Pei F., Wu L., Zhang Y., Liao Y., Kang Q., Han Y., Zhang H., Shen Y., Xu H., Li Z. (2024). Interfacial Self-Healing Polymer Electrolytes for Long-Cycle Solid-State Lithium-Sulfur Batteries. Nat. Commun..

[83] Wang Y., Xu Z., Affinito J.D., Skaggs C.D. (2010). Primer for Battery Electrode. U.S. Patent.

[84] Mecerreyes D., Casado N., Villaluenga I., Forsyth M. (2024). Current Trends and Perspectives of Polymers in Batteries. Macromolecules.

[85] Cui P., Zhang Q., Sun C., Gu J., Shu M., Gao C., Zhang Q., Wei W. (2022). High Ion Conductivity Based on a Polyurethane Composite Solid Electrolyte for All-Solid-State Lithium Batteries. RSC Adv..

[86] He T., Ding Y., Zhang H., Liu C., Lou X., Zhu S., Yang X., Yang L., Bai H. (2025). Self-Healing SA@ Borax Binder for In Situ Tuning of the Solid Electrolyte Interfaces for Silicon Anodes. ACS Sustain. Chem. Eng..

[87] Chen J., Wang F., Zheng J., Wang J., Li W., Zhang J., Zhang L., Chen J. (2024). An interweaving 3D ion-conductive network binder enabling stable aqueous rechargeable batteries. J. Mater. Chem. A.

[88] Mathew A., van Ekeren W., Andersson R., Lacey M.J., Heiskanen S.K., Younesi R., Brandell D. (2024). Limitations of Polyacrylic Acid Binders When Employed in Thick LNMO Li-Ion Battery Electrodes. J. Electrochem. Soc..

[89] Nagulapati V.M., Kim D.S., Oh J., Lee J.H., Hur J., Kim I.T., Lee S.G. (2019). Enhancing the Electrochemical Performance of SbTe Bimetallic Anodes for High-Performance Sodium-Ion Batteries: Roles of the Binder and Carbon Support Matrix. Nanomaterials.

[90] Zhang R., Yang X., Zhang D., Qiu H., Fu Q., Na H., Guo Z., Du F., Chen G., Wei Y. (2015). Water Soluble Styrene Butadiene Rubber and Sodium Carboxyl Methyl Cellulose Binder for ZnFe_2_O_4_ Anode Electrodes in Lithium Ion Batteries. J. Power Sources.

[91] Isozumi H., Horiba T., Kubota K., Hida K., Matsuyama T., Yasuno S., Komaba S. (2020). Application of Modified Styrene-Butadiene-Rubber-Based Latex Binder to High-Voltage Operating LiCoO_2_ Composite Electrodes for Lithium-Ion Batteries. J. Power Sources.

[92] Park K., Yoo H.E., Jung Y., Ryu M., Myeong S., Lee D., Kim S.C., Kim C., Kim J., Kwon J. (2023). Styrene-Butadiene Rubber Patterned Current Collector for Improved Li-Ion Kinetics of the Anode for High Energy Density Lithium-Ion Batteries. J. Power Sources.

[93] Liao K.Y., Chang C.C., Lee Y.L., Wen T.C. (2025). Applying Carboxymethyl Cellulose-Based Aqueous Binder with Zwitterion Molecules in Graphite Anode for Lithium-Ion Batteries. Appl. Surf. Sci..

[94] Dhanumalayan E., Joshi G.M. (2018). Performance Properties and Applications of Polytetrafluoroethylene (PTFE)—A Review. Adv. Compos. Hybrid Mater..

[95] Gao S., Su Y., Bao L., Li N., Chen L., Zheng Y., Tian J., Li J., Chen S., Wu F. (2015). High-Performance LiFePO_4_/C Electrode with Polytetrafluoroethylene as an Aqueous-Based Binder. J. Power Sources.

[96] Bigoni F., De Giorgio F., Soavi F., Arbizzani C. (2017). Sodium Alginate: A Water-Processable Binder in High-Voltage Cathode Formulations. J. Electrochem. Soc..

[97] Balasooriya W., Schrittesser B., Pinter G., Schwarz T., Conzatti L. (2019). The Effect of the Surface Area of Carbon Black Grades on HNBR in Harsh Environments. Polymers.

[98] Verdier N., El Khakani S., Lepage D., Prébé A., Aymé-Perrot D., Dollé M., Rochefort D. (2019). Polyacrylonitrile-Based Rubber (HNBR) as a New Potential Elastomeric Binder for Lithium-Ion Battery Electrodes. J. Power Sources.

[99] Majeed S., Rasheed T., Shafi S., Bagheri A.R., Nguyen T.A., Haq N.U., Bilal M. (2021). Waterborne Polyurethane-Based Electrode Nanomaterials. Biopolymeric Nanomaterials: Fundamentals and Applications.

[100] Zhu C.L., Tao C., Bao J.J., Huang Y.P., Xu G.W. (2015). Waterborne Polyurethane Used as Binders for Lithium-Ion Battery with Improved Electrochemical Properties. Adv. Mater. Res..

[101] Zheng M., Cai X., Tan Y., Wang W., Wang D., Fei H., Saha P., Wang G. (2020). A High-Resilience and Conductive Composite Binder for Lithium-Sulfur Batteries. Chem. Eng. J..

[102] Eom J.Y., Kim S.I., Ri V., Kim C. (2019). The Effect of Polymeric Binders in the Sulfur Cathode on the Cycling Performance for Lithium-Sulfur Batteries. Chem. Commun..

[103] Yang M., Rong Z., Li X., Yuan B., Zhang W. (2025). Zwitterionic Polymer as Binder for LiFePO_4_ Cathodes in Lithium-Ion Batteries. Chem. Eng. J..

[104] Park G.G., Park Y.K., Park J.K., Lee J.W. (2017). Flexible and Wrinkle-Free Electrode Fabricated with Polyurethane Binder for Lithium-Ion Batteries. RSC Adv..

[105] Nguyen V.H., Wang W.L., Jin E.M., Gu H.B. (2013). Impacts of Different Polymer Binders on Electrochemical Properties of LiFePO_4_ Cathode. Appl. Surf. Sci..

[106] Bhattarai S., Lee S.I., Lee D.S., Lee Y.S. (2019). Effect of Molecular Weight of Poly(Tetramethylene Glycol) on Waterborne Polyurethane Dispersion Coating Gloss. Bull. Korean Chem. Soc..

[107] Lv P., Liu H., Cui Z., An H., Yang M., Xie S., Sun X., Tang C. (2025). Covalently Cross-Linked Waterborne Polyurethane Acrylate Network Binder for Low-Expansion Silicon/Carbon Anode. J. Power Sources.

[108] Lv Z., Li T., Hou X., Wang C., Zhang H., Yan J., Zheng Q., Li X. (2022). Solvation structure and solid electrolyte interface engineering for excellent Na+ storage performances of hard carbon with the ether-based electrolytes. Chem. Eng. J..

[109] Yu Y., Lu F., Sun N., Wu A., Pan W., Zheng L. (2018). Single Lithium-Ion Polymer Electrolytes Based on Poly(Ionic Liquid)s for Lithium-Ion Batteries. Soft Matter.

[110] Zhang M., Zhao F., Luo Y. (2019). Self-Healing Mechanism of Microcracks on Waterborne Polyurethane with Tunable Disulfide Bond Contents. ACS Omega.

[111] Vu V.-P., So H.-M., Kim A., Lee J.Y., Oh M., Hyun S. (2025). Self-Healing Polymer Binders: Next-Generation Battery Applications. J. Mater. Chem. A Mater..

[112] Oya N., Ikezaki T., Yoshie N. (2013). A Crystalline Supramolecular Polymer with Self-Healing Capability at Room Temperature. Polym. J..

[113] Chen T., Shao M., Zhang Y., Zhang X., Xu J., Li J., Wang T., Wang Q. (2024). Ultratough Supramolecular Polyurethane Featuring an Interwoven Network with Recyclability, Ideal Self-Healing and Editable Shape Memory Properties. ACS Appl. Mater. Interfaces.

[114] Banan A.R., Keshavarz S.M. (2025). Biobased Self Healing Waterborne Polyurethane with Vanillin Derived Dynamic Imine Bonds for Enhanced Mechanical Strength and Performance. Sci. Rep..

[115] Sathya S., Soosaimanickam C., Bella F., Yoo D.J., Stephan A.M. (2024). Cycling Performance of SiOx-Si-C Composite Anode with Different Blend Ratios of PAA-CMC as Binder for Lithium Sulfur Batteries. J. Polym. Res..

[116] Peng B., Liu D., Ji M., Liu Y., Liao X., Chen J., Qiu L., Qu D. (2025). Low-Cost and Multifunctional Copolymer Binder for Stabilizing High-Capacity Si/C Composite Anodes in Practical Lithium-Ion Batteries. ACS Appl. Energy Mater..

[117] Zhou X., Zhou Y., Yu L., Qi L., Oh K.S., Hu P., Lee S.Y., Chen C. (2024). Gel Polymer Electrolytes for Rechargeable Batteries toward Wide-Temperature Applications. Chem. Soc. Rev..

[118] Tang C., Yu L., Jiang Q., Gu R., Zhang Y., Liu Z., Cai W., Wu H., Zhang Y., Yao M. (2024). Polymer-Based Electrolyte for Lithium-Based High-Energy-Density and Safe Energy Storages Devices: Strategy and Mechanisms. Renewables.

[119] Xi G., Xiao M., Wang S., Han D., Li Y., Meng Y. (2021). Polymer-Based Solid Electrolytes: Material Selection, Design, and Application. Adv. Funct. Mater..

[120] An Y., Han X., Liu Y., Azhar A., Na J., Nanjundan A.K., Wang S., Yu J., Yamauchi Y. (2022). Progress in Solid Polymer Electrolytes for Lithium-Ion Batteries and Beyond. Small.

[121] Li Z., Fu J., Zhou X., Gui S., Wei L., Yang H., Li H., Guo X. (2023). Ionic Conduction in Polymer-Based Solid Electrolytes. Adv. Sci..

[122] Jun S.Y., Shin K., Lee J.S., Kim S., Chun J., Ryu W.H. (2023). Molecular Dipoles as a Surface Flattening and Interface Stabilizing Agent for Lithium-Metal Batteries. Adv. Sci..

[123] Ordaz M.V., Pavlin N., Gastaldi M., Gerbaldi C., Dominko R. (2024). Protective Coating for Stable Cycling of Li-Metal Batteries Based on Cellulose and Single-Ion Conducting Polymer. ACS Appl. Mater. Interfaces.

[124] Gao J., Wang C., Han D.-W., Shin D.-M. (2021). Single-ion conducting polymer electrolytes as a key jigsaw piece for next-generation battery applications. Chem. Sci..

[125] Meng P., Yang Z., Jiang M., Zhao T., Zhang J., Fu C. (2024). Engineering Ternary Hydrated Eutectic Electrolytes to Realize Rechargeable Cathode-Free Aluminum-Ion Batteries. Energy Storage Mater..

[126] Zhang D., Meng X., Hou W., Hu W., Mo J., Yang T., Zhang W., Fan Q., Liu L., Jiang B. (2023). Solid Polymer Electrolytes: Ion Conduction Mechanisms and Enhancement Strategies. Nano Res. Energy.

[127] Han S., Hu Z., Zhang W., Hu J., Yang L. (2022). Flexible Segments Regulating the Gelation Behaviours of Aliphatic Polycarbonate Gels with Excellent Shape Memory and Self-Healing Properties. J. Mol. Liq..

[128] Daems K., Yadav P., Dermenci K.B., Van Mierlo J., Berecibar M. (2024). Advances in Inorganic, Polymer and Composite Electrolytes: Mechanisms of Lithium-Ion Transport and Pathways to Enhanced Performance. Renew. Sustain. Energy Rev..

[129] Ram Prasanth S., Prasannavenkadesan V., Katiyar V., Achalkumar A.S. (2025). Polymer Electrolytes: Evolution, Challenges, and Future Directions for Lithium-Ion Batteries. RSC Appl. Polym..

[130] Tang L., Chen B., Zhang Z., Ma C., Chen J., Huang Y., Zhang F., Dong Q., Xue G., Chen D. (2023). Polyfluorinated Crosslinker-Based Solid Polymer Electrolytes for Long-Cycling 4.5 V Lithium Metal Batteries. Nat. Commun..

[131] Zhou Z., Yuan Y., Tang X., Liu W., Yang B., Wen J., Liu L. (2025). Enhancing Ionic Conductivity of Polyethylene Oxide-Based Solid-State Electrolytes through Blending with a Small Amount of Polyacrylic Acid: A Polymer-Anion Synergistic Mechanism. J. Power Sources.

[132] Bao J., Qu X., Qi G., Huang Q., Wu S., Tao C., Gao M., Chen C. (2018). Solid electrolyte based on waterborne polyurethane and poly(ethylene oxide) blend polymer for all-solid-state lithium ion batteries. Solid State Ion..

[133] Cheng T.T., Wen T.C. (1998). Novel Water-Borne Polyurethane Based Electrolytes for Lithium Batteries—(I) Tailor-Made Polymer. J. Electroanal. Chem..

[134] Yang C.H., Lin W.C., Liu F.J. (2007). Waterborne Polyurethane Single-Ion Electrolyte from Aliphatic Diisocyanate and Various Molecular Length of Polyethylene Glycol. Express. Polym. Lett..

[135] Shi Z., Zhou H., Fan Z., Guo K., Nie H., Zhou X., Xue Z. (2024). Waterborne Polyurethane Micelles Reinforce PEO-Based Electrolytes for Lithium Metal Batteries. Small.

[136] Fernández M.E., Diosa J.E., Vargas R.A., De Los J., Villaquiran C.F., Garcia D., Eiras J.A. (2007). New Polymer Electrolyte Based on PVAL-LiOH-Al_2_O_3_-H_2_O. Phys. Status Solidi (C) Curr. Top. Solid State Phys..

[137] Wen T.-C., Du Y.-L., Digar M. (2002). Compositional effect on the morphology and ionic conductivity of thermoplastic polyurethane based electrolytes. Eur. Polym. J..

[138] Meng X., Du M., Li Y., Du S., Zhao L., Zheng S., Zhang J., Li H., Qiao L., Tan K.B. (2024). Solidify Eutectic Electrolytes via the Added MXene as Nucleation Sites for a Solid-State Zinc-Ion Battery with Reconstructed Ion Transport. Nano Lett..

[139] Sarfraz N., Kanwal N., Ali M., Ali K., Hasnain A., Ashraf M., Ayaz M., Ifthikar J., Ali S., Hendi A. (2024). Materials Advancements in Solid-State Inorganic Electrolytes for Highly Anticipated All Solid Li-Ion Batteries. Energy Storage Mater..

[140] Lee M.-H., Dam T., Baek S.-J., Park C.-J. (2025). Li_2_SO_4_-Infused SiO_2_ Filler-Based in-Situ Polymerized Composite Electrolytes for Solid-State Lithium Metal Batteries. J. Power Sources.

[141] Zhao Y., Yan J., Cai W., Lai Y., Song J., Yu J., Ding B. (2019). Elastic and Well-Aligned Ceramic LLZO Nanofiber Based Electrolytes for Solid-State Lithium Batteries. Energy Storage Mater..

[142] Asghar M.R., Anwar M.T., Naveed A., Zhang J. (2019). A Review on Inorganic Nanoparticles Modified Composite Membranes for Lithium-Ion Batteries: Recent Progress and Prospects. Membranes.

[143] Tang S., Guo W., Fu Y. (2021). Advances in Composite Polymer Electrolytes for Lithium Batteries and Beyond. Adv. Energy Mater..

[144] Handayani P.L., Kim G., Cho A., Jeon Y., Kim N., Kim S., Ahn S., Jeong H., Choi U.H. (2025). Uniform and high Li^+^ transporting polymer electrolytes for stable and long-cycle-life lithium metal batteries: Current status and future. Small.

[145] Cheng Y.T., Wen T.C. (1998). Novel Waterborne Polyurethane Based Electrolytes for Lithium Batteries—(II) the Effect of Adding LiCF_3_SO_3_–PC. Solid State Ion..

[146] Cui P., Sun C., Wei W. (2023). Polyurethane/Li_10_GeP_2_S_12_ composite electrolyte with high ions transfer number and ions capture for all-solid-state lithium batteries. Energy Mater..

[147] Luo J., Zhang Q. (2024). In Situ Polymer Gel Electrolyte in Boosting Scalable Fibre Lithium Battery Applications. Nano-Micro Lett..

[148] Zhong J., Wang Z., Yi X., Li X., Guo H., Peng W., Wang J., Yan G. (2024). Breaking the Solubility Limit of LiNO_3_ in Carbonate Electrolyte Assisted by BF3 to Construct a Stable SEI Film for Dendrite-Free Lithium Metal Batteries. Small.

[149] Jin Q., Sun X., Chen H., Luo Q., Mo M., Wen C., Huang L., Lu Z., Qing N., Tang L. (2025). Self-Matting Waterborne Polyurethane Coatings With Ultra-Low Gloss and Enhanced Corrosion Resistance via Molecular Design and ZnO Integration. Polym. Adv. Technol..

[150] Gogia A., Wang Y., Rai A.K., Bhattacharya R., Subramanyam G., Kumar J. (2021). Binder-Free, Thin-Film Ceramic-Coated Separators for Improved Safety of Lithium-Ion Batteries. ACS Omega.

[151] Pan Y., Chou S., Liu H.K., Dou S.X. (2017). Functional Membrane Separators for Next-Generation High-Energy Rechargeable Batteries. Natl. Sci. Rev..

[152] Choi H., Lee B.S. (2022). Pilot Scale Hybrid Organic/Inorganic Coatings on a Polyolefin Separator to Enhance Dimensional Stability for Thermally Stable Long-Life Rechargeable Batteries. Polymers.

[153] Ying D., Chen K., Lu J., Wu C., Chen B., Lv Y., Liu Y., Fang Z. (2025). Aramid and Zirconia Coated Separator for Enhanced Electrochemical Performance of Lithium-Ion Batteries. RSC Adv..

[154] Kanamura K., Munakata H., Jin Y. (2014). Lithium Secondary Battery Separator and Method of Manufacturing Same. U.S. Patent.

[155] Kim T.H., Kim M.S., Kim E.J., Ju M., Kim J.S., Lee S.H. (2024). Highly Stretchable Thermoplastic Polyurethane Separators for Li-Ion Batteries Based on Non-Solvent-Induced Phase Separation Method. Polymers.

[156] Zhang J., Zhai R., Kang W., Li J., Hong C., Xu Y., Zhou C. (2025). Construction of Waterborne Polyurethane Crosslinking Network with Excellent Adhesion Strength, Mechanical Properties, and Wear Resistance. Mater. Today Commun..

[157] Noh J.-H., Son H.-J., Lee H.-C., Park J.-H. (2019). A Study on High Thermal Stable Separator Coating Machine for High-Capacity Lithium Ion Secondary Battery. J. Korean Soc. Manufac. Process Eng..

[158] Liu Z., Zhou X., Wu Z., Gao C., Liu Y., Wang Y., Sun L., Ding L. (2025). Preparation of Waterborne Polyurethane Coatings with Enhanced Mechanical Properties and Superhydrophobic Surface. Mater. Chem. Phys.

[159] Hu S., Lin S., Tu Y., Hu J., Wu Y., Liu G., Li F., Yu F., Jiang T. (2016). Novel Aramid Nanofiber-Coated Polypropylene Separators for Lithium Ion Batteries. J. Mater. Chem. A Mater..

[160] Li H., Jia R., Chen W., Wei C., Ji Q., Long X., Wang B., Zhang K., Feng J., Tan L. (2025). Bio-based separator engineering toward better metal anodes in rechargeable batteries: Progress and perspectives. Adv. Funct. Mater..

